# Dual-Mode Sensor with Saturated Mechanochromic Structural Color Enhanced by Black Conductive Hydrogel for Interactive Rehabilitation Monitoring

**DOI:** 10.1007/s40820-025-01963-2

**Published:** 2026-01-11

**Authors:** Zhiyuan Sun, Binhong Yu, Chao Dong, Chengjun Yu, Lianghe Sheng, Zhe Cui, Yaming Liu, Zhenni Lu, Bingda Chen, Daixi Xie, Zhandong Huang, Songshan Zeng, Qingdong Ou

**Affiliations:** 1 Macao Institute of Materials Science and Engineering (MIMSE), Faculty of Innovation Engineering, Macau University of Science and Technology, Taipa, Macao, 999078 People’s Republic of China; 2 Macau University of Science and Technology Zhuhai MUST Science and Technology Research Institute, Zhuhai, 519031 People’s Republic of China; 3 Chemistry and Physics Department, College of Art and Science, The University of Texas of Permian Basin, Odessa, TX 79762 USA; 4 Department of Oncology, The First Affiliated Hospital of Jinan University, Guangzhou, 510630 People’s Republic of China; 5 Key Laboratory of Green Printing, Institute of Chemistry, Chinese Academy of Sciences, Beijing Engineering Research Center of Nanomaterials for Green Printing Technology, Beijing National Laboratory for Molecular Sciences (BNLMS), Zhongguancun North First Street 2, Beijing, 100190 People’s Republic of China; 6 School of Chemical Engineering and Technology, Xi’an Jiaotong University, Xi’an, 710049 People’s Republic of China

**Keywords:** Conductive hydrogel, Structural color, Hydroxypropyl cellulose, Dual-mode sensor, Rehabilitation monitoring

## Abstract

Flexible and wearable sensors offer immense potential for rehabilitation medicine, but most rely solely on electrical signals, lacking real-time visual feedback and limiting trainee’s interactivity. Inspired by the structural coloration of Cyanocitta stelleri feathers, we developed a dual-mode sensor by utilizing black conductive polymer hydrogel (CPH)-enhanced structural color strategy. This sensor integrates a hydroxypropyl cellulose (HPC)-based structural color interface with a designed CPH sensing component. Highly visible light-absorbing CPH (absorption rate > 88%) serves as the critical substrate for enhancing structural color performance. By absorbing incoherent scattered light and suppressing background interference, it significantly enhances the saturation of structural color, thereby achieving a high contrast index of 4.92. Unlike the faint and hardly visible structural colors on non-black substrates, the HPC on CPH displays vivid, highly perceptible colors and desirable mechanochromic behavior. Moreover, the CPH acts as a flexible sensing element, fortified by hydrogen and coordination bond networks, and exhibits exceptional electromechanical properties, including 867.1 kPa tensile strength, strain sensitivity (gauge factor of 4.24), and outstanding durability (over 4400 cycles). Compared to traditional single-mode sensors, the integrated sensor provides real-time visual and digital dual feedback, enhancing the accuracy and interactivity of rehabilitation assessments. This technology holds promise for advancing next-generation rehabilitation medicine.

**Supplementary Information:**

The online version contains supplementary material available at 10.1007/s40820-025-01963-2.

## Highlights

- Mimicking Cyanocitta stelleri feathers, we developed a dual-mode sensor with strain-sensing and mechanochromic functions by using black conductive polymer hydrogel (CPH) substrate to enhance hydroxypropyl cellulose’s structural color.
- The synthesized CPH, with >88% visible-light absorption, enhances color saturation by absorbing scattered light and suppressing background interference, enabling vivid mechanochromism. Fortified by noncovalent bonds, it also functions as a robust, sensitive sensor.
- Unlike traditional single-mode sensors, this integrated sensor offers real-time visual and digital feedback, improving rehabilitation assessment accuracy and interactivity.

**Supplementary Information:**

The online version contains supplementary material available at 10.1007/s40820-025-01963-2.

## Graphical abstract

**Figure:**
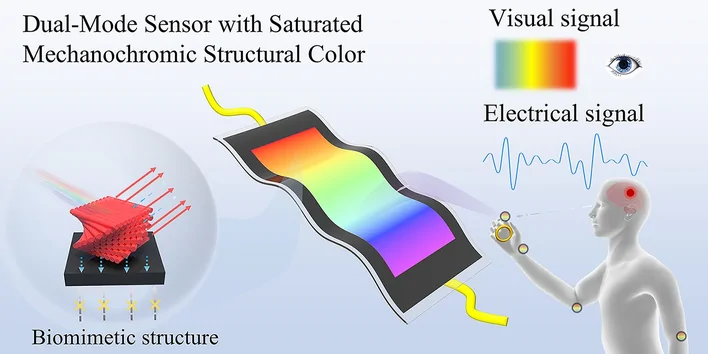

## Introduction

Stroke is the cerebrovascular disease with the highest disability rate worldwide, leaving millions of new patients with mobility impairments each year, creating substantial economic burdens for families and societies globally [1, 2]. Fortunately, systematic rehabilitation training can reactivate the central nervous system’s regulatory mechanism for motor function, helping patients gradually regain mobility and improve their quality of life [3, 4]. Notably, the effectiveness of rehabilitation mechanisms based on neural remodeling largely depends on precise quantification and real-time feedback of joint range of motion during rehabilitation [5]. However, conventional clinical assessment tools, such as the Fugl-Meyer Assessment scale, predominantly rely on subjective physician evaluations, lacking the capacity to provide accurate and objective individualized data [6, 7]. Furthermore, electronic devices like surface electromyography suffer from mechanical mismatch between rigid electrodes and human skin/tissues, which hinders continuous monitoring over the months-long rehabilitation period [8, 9]. Consequently, these conventional evaluation approaches are often unable to align with patients’ actual rehabilitation trajectories, potentially compromising therapeutic outcomes and prolonging functional recovery periods.

Flexible sensors have shown great potential in personalized health care and rehabilitation monitoring, driven by advances in wearable electronics and Internet of Things technologies [10–15]. Among various flexible sensor materials, conductive polymer hydrogels (CPHs) stand out as ideal materials for intelligent rehabilitation systems, merging the softness of hydrogels with the electrical conductivity of conductive polymers [16–21]. Currently, CPH sensors based on polyaniline, polypyrrole, and poly (3,4-ethylene dioxythiophene) can convert mechanical stimuli into easily detectable electrical signals, finding successful applications in health monitoring scenarios such as motion perception, sweat analysis, temperature sensing, and electrophysiological signal monitoring [22, 23]. Moreover, through molecular design and structural engineering, researchers have further endowed these materials with advanced functionalities such as self-healing capability, tissue adhesion, anti-swelling behavior, and fatigue resistance, significantly improving the comfort and stability of the CPH sensors [24]. Nevertheless, CPH sensors still face substantial challenges in practical applications. First, the thermodynamic mismatch between hydrophobic conductive polymers and hydrophilic hydrogel matrices leads to poor compatibility, weakening interfacial bonding and triggering a trade-off between conductivity and mechanical properties [25]. Second, most CPH sensors predominantly rely on electrical signal changes to reflect motion states, necessitating specialized equipment for data interpretation, fundamentally limiting their capacity to satisfy real-time interaction requirements for rehabilitation therapies [26]. An ideal rehabilitation sensor should integrate high-fidelity electrical signal acquisition with intuitive visual feedback mechanisms, thereby enabling real-time training quality assessment and positive feedback for trainees. Therefore, developing novel sensors integrating excellent mechanical properties, high electrical conductivity, and real-time visual feedback functions represents a critical imperative for advancing next-generation sensing technologies.

Hydroxypropyl cellulose (HPC), an environmentally friendly cellulose derivative, has emerged as a promising candidate material for intelligent sensing applications due to its abundant sources, excellent biocompatibility, and unique stimulus-responsive properties [27, 28]. When dissolved in an aqueous solution above a critical concentration, HPC molecules spontaneously assemble through intermolecular hydrogen bonding into a liquid crystal with a periodic helical structure [29]. This unique helical stacking architecture enables selective reflection of specific light wavelengths via the Bragg reflection mechanism, producing vivid structural colors with metallic luster. Notably, the pitch of the HPC helical structure undergoes dynamic reconstruction in response to external stimuli such as temperature, humidity, or mechanical stress, thereby achieving a dynamic structural color response [30, 31]. However, the HPC’s achromatic nature and absence of intrinsic light-absorbing components render its optical signals highly susceptible to background interference [32, 33]. Particularly in multi-color backgrounds, incoherent scattering-induced spectral interference substantially compromises color fidelity and contrast resolution, posing critical challenges for naked-eye detection applications [34]. Consequently, developing HPC-based vision sensors with high color saturation remains a significant challenge.

Herein, inspired by the mechanism of synergistic coloration in Cyanocitta stelleri’s plumage, we proposed an innovative strategy based on black CPH-enhanced structural color (BCESC) to develop a dual-mode sensing system, designated as DM-BCESC sensor. The DM-BCESC sensor integrates a HPC structural color component with a CPH, achieving strain sensing and mechanochromic interaction functionalities. In this device, the CPH sensing layer acts not only as a flexible conductive component but also as a key substrate for enhancing the structural color of HPC. CPH is a synthetic polyacrylamide–sodium alginate–polyaniline (PAM–ALG–PANI) black hydrogel with high light absorption (absorption rate > 88%) across the entire visible light spectrum, which enhances the color saturation of the HPC, providing optical support for structural color visualization. Moreover, by incorporating hydrogen and coordination bonds into the hydrogel network, the CPH with excellent electromechanical properties was developed. DFT calculations verified that these non-covalent bonds improve interfacial interaction between the hydrophobic conductive polymers and hydrophilic hydrogel matrices. The obtained hydrogel demonstrates excellent electromechanical performance, featuring a tensile strength of 867.1 kPa, alongside high electrical conductivity (4.08 S m⁻^1^) and remarkable strain sensitivity (GF = 4.24). The structural color top layer is composed of HPC liquid crystals with a cholesteric phase, which undergo reversible structural color changes under external stimuli, serving as a visual feedback interface. Notably, the black PAM–ALG–PANI hydrogel plays a pivotal role in optimizing structural color saturation. Experimental evidence reveals that HPC on non-black substrates generates only faint structural colors barely perceptible by human vision. In contrast, when supported on the black PAM–ALG–PANI hydrogel substrate, HPC develops vivid structural colors with high visual perceptibility. To quantify color performance, a contrast index is introduced as an evaluation parameter, defined as the ratio of structural color reflectance to background substrate reflectance. HPC on black CPH exhibits a contrast index of 4.92, which is over 5.7 times higher than those on non-black substrates. The integrated DM-BCESC sensor combines electrical signal-based motion tracking with mechanochromic structural coloration, delivering both real-time visual feedback and digital precision in rehabilitation monitoring. This synergistic visual–electrical feedback system overcomes functional limitations inherent in conventional single-mode sensors, demonstrating promising potential in sports rehabilitation, intelligent human–machine interfaces, and wearable electronics.

## Experimental Section

### Materials

Acrylamide (AAm, 99%), N, N’-methylene-bis-acrylamide (MBAA, 99%), tetramethylethylenediamine (TEMED, 99%), ammonium persulfate (APS, 99%), sodium alginate (ALG, viscosity 200 ± 20 mpa s), aniline (ANI, ≥ 99.9%), and iron (III) nitrate (Fe (NO_3_)_3_, 99.9%) were purchased from Macklin (Shanghai, China). Hydrochloric acid (HCl, 36.0 − 38.0%) was obtained from Titan (Shanghai, China). Hydroxypropyl cellulose was supplied by NISSO Chemical Europe (HPC, Food grade, Mw 40,000 g mol^−1^). Deionized water (DI) was made in the laboratory.

### Preparation of PAM–ALG Hydrogels

Initially, 42 g of acrylamide (AAm) and 7 g of alginate (ALG) were dissolved in 300 mL of distilled water under continuous stirring. Subsequently, 0.024 g of N, N’-methylenebisacrylamide (MBAA), 0.048 g of ammonium persulfate (APS), and 80 μL of N, N, N’, N’-tetramethylethylenediamine (TEMED) were introduced into 40 mL of the previously prepared solution with vigorous stirring to ensure thorough mixing. The resulting mixture was then promptly transferred into plastic molds and subjected to polymerization at 50 °C for 5 h, leading to the formation of polyacrylamide–alginate (PAM–ALG) hydrogels.

### Synthesis of PAM–ALG–PANI Hydrogels

First, ANI solutions with varying concentrations (1, 2, 3, and 4 wt.%) were prepared by mixing equal amounts of ANI and HCl in distilled water. Subsequently, PAM–ALG hydrogels were immersed in ANI solutions for 6 h to facilitate the absorption of the ANI monomer. Then, the hydrogels were transferred to a 10 wt.% Fe (NO_3_)_3_ solution to induce further polymerization of the ANI within the hydrogel matrix, resulting in the synthesis of PAM–ALG–PANI hydrogels. The obtained hydrogel was washed with deionized water.

### Preparation of HPC Cholesteric Liquid Crystal (CLC)

HPC mesophases were prepared by blending HPC and deionized water at a weight ratio of 3:1.65. After vigorous stirring, the samples were degassed through centrifugation and subsequently stored at room temperature for 2 days until coloration occurred.

### Fabrication of Dual-Mode Sensors

The PAM–ALG–PANI hydrogel linking the wires was enclosed by two transparent pieces of VHB tape serving as the electrode layer. The gaps at the seams of the VHB tape were sealed with silicone to prevent leakage or external interference. Subsequently, VHB tapes with varied patterns were affixed onto the electrode layer’s VHB tapes to act as templates for HPC filling. The HPC CLC was then introduced into the VHB templates and sealed with another transparent VHB piece. The resulting devices were left at room temperature for 30 min to induce coloration.

### Characterization

Chemical bonding analysis was employed using attenuated total reflectance Fourier transform infrared spectroscopy (Bruker VERTEX 70 spectrometer). Molecular vibration characteristics were investigated through micro-confocal Raman spectroscopy (HORIBA LabRAM HR Evolution) employing a 532 nm excitation laser, with spectral data collected across 500 − 2000 cm⁻^1^. For mechanical characterization, a Xinke XK-509 universal testing system was utilized to assess tensile and compressive properties. Crystalline structures were visualized through polarized optical microscopy (AOSVI M330P-AF 1200). Optical absorption and reflection profiles were recorded using a NOVA2S-EX fiber optic spectrometer. Electrical conductivity measurements were taken through four-point probe methodology using a Jingge ST2242 system. Cell viability was quantitatively assessed using the CCK-8 assay with absorbance measured at 450 nm via a micro-plate reader (Thermo Fisher Scientific, JQ58018) and qualitatively evaluated through Calcein AM/PI staining observed with an inverted fluorescence microscope. Real-time electromechanical responses during deformation were monitored with a Tonghui TH2832 LCR Digital Bridge. To ensure measurement accuracy, all electrical tests were performed on the material in an encapsulated setup to isolate it from ambient conditions.

### Quantum Mechanic Calculations

Gas-phase geometry optimizations, numerical frequency calculations, were performed at the density functional theory (DFT) level using the Gaussian 16 software packages. All Gaussian calculations used the B3LYP hybrid exchange correlation functional. A 6–311 +  + g** basis set was used for all atoms except for Fe where a LANL2DZ basis set, which included an effective core potential, was used. The vibrational frequencies were then calculated without negative value based on this optimized geometry to make sure it is at global minima.

## Results and Discussion

### Design Mechanism for DM-BCESC Sensors

**Fig. 1:**
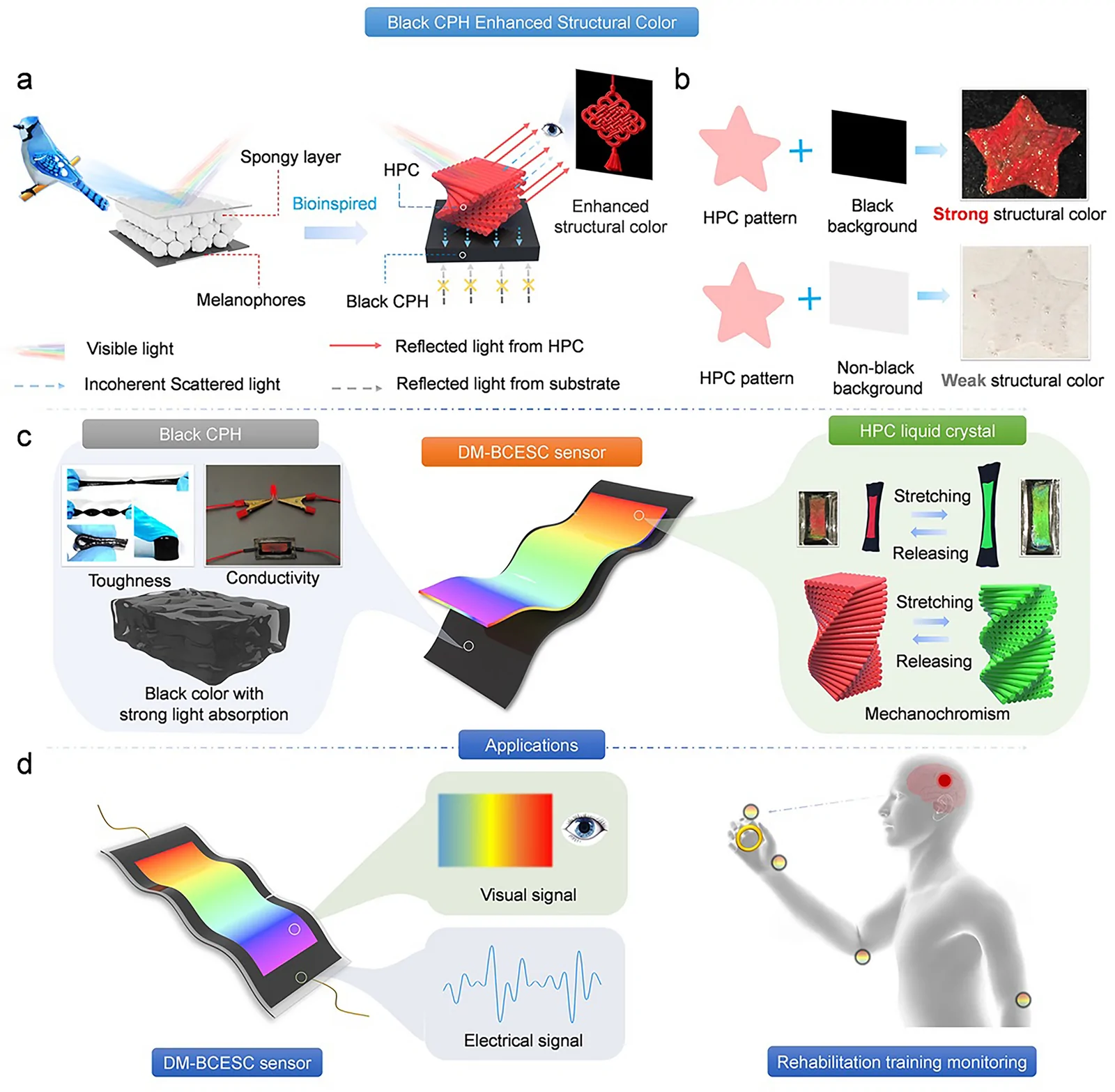
Design and applications of the DM-BCESC sensor. **a** Design of a DM-BCESC sensor with structural color enhancement properties, inspired by the blue feather structure of Cyanocitta stelleri. **b** Comparative photographs showcasing structural color patterns on a black background (top) and a non-black background (bottom). **c** Composition and characteristics of the DM-BCESC sensor. **d** Functions and applications of the DM-BCESC sensor

In nature, avian species employ sophisticated coloration strategies to fulfill critical ecological functions such as visual communication, mate attraction, and survival adaptation [35]. The remarkable plumage coloration observed in birds primarily originates from intricate biological structures rather than pigmental effects alone [32]. A representative example is the Cyanocitta stelleri, whose iconic cobalt-blue feathers originate from a specialized three-layered architecture, including an outer translucent keratin sheath, a middle spongy matrix containing nanoscale air cavities, and an underlying melanin-rich layer [36]. When incident light penetrates the keratin sheath into the spongy layer, the periodically arranged air sacs induce constructive interference, selectively reflecting blue wavelengths. Notably, the spongy layer alone produces only white feathers with a faint bluish tinge. This is because melanin also plays an integral role in the blue coloring process, absorbing incoherently scattered white light from the sponge layer, thereby increasing the purity of the color produced by the sponge layer [36, 37]. Inspired by this multilayered synergistic color-rendering mechanism, we develop a DM-BCESC sensor by using black CPH as a substrate to enhance the structural color of the HPC layer (Fig. 1a). The CPH with broadband light-absorbing capability achieves color enhancement through two synergistic pathways, operating via a principle analogous to melanin in avian feathers. First, this black matrix effectively suppresses incoherent scattered light of different wavelengths, minimizing optical interference that can weaken the structural color signal. Second, the dark substrate inherently suppresses background reflections from underlying layers, thereby enhancing color saturation by eliminating competing light paths. Visualization results demonstrate that the red HPC star-shaped pattern on the black CPH substrate exhibits sharp boundaries and highly saturated coloration, whereas the same pattern on non-black substrates only shows faint and blurred outlines with weak structural color (Fig. 1b). These observations confirm the critical role of black CPH in enhancing structural color performance of HPC.

As shown in Fig. 1c, the DM-BCESC sensor is composed of two functional layers, including a strain-sensitive base layer and an upper mechanochromic interface. The strain sensing layer, made of mechanically flexible and electrically conductive CPH, converts structural deformations into electrical signal changes under complex stress conditions, thus ensuring accurate and reliable detection of mechanical signals. Meanwhile, its dark substrate significantly enhances chromatic contrast for the mechanochromic interface, providing optical support for visual feedback. The visual interface is made of HPC liquid crystal with a cholesteric phase structure, which exhibits structural colors caused by Bragg reflection. When subjected to mechanical deformation, the pitch of HPC liquid crystals undergoes reversible reorganization, producing macroscopic color shifts across the visible spectrum [30]. This mechanochromic property enables real-time motion feedback through structural color changes that are easily discernible to the naked eye, eliminating the need for specialized equipment. In stroke rehabilitation monitoring applications, the DM-BCESC sensing system achieves synergistic feedback through concurrent electrical signal detection and visual interaction (Fig. 1d). The HPC layer’s structural color shifts provide real-time visual interaction, enabling trainees to self-adjust movements. Simultaneously, the CPH layer can accurately quantify joint motion parameters, such as movement amplitude and frequency. This collaborative feedback mechanism not only enhances patients’ motivation through color changes but also provides digital evaluation criteria via electrical signals, showcasing significant application potential in the field of rehabilitation medicine.

### Synthesis and Characterization of Black CPHs

**Fig. 2:**
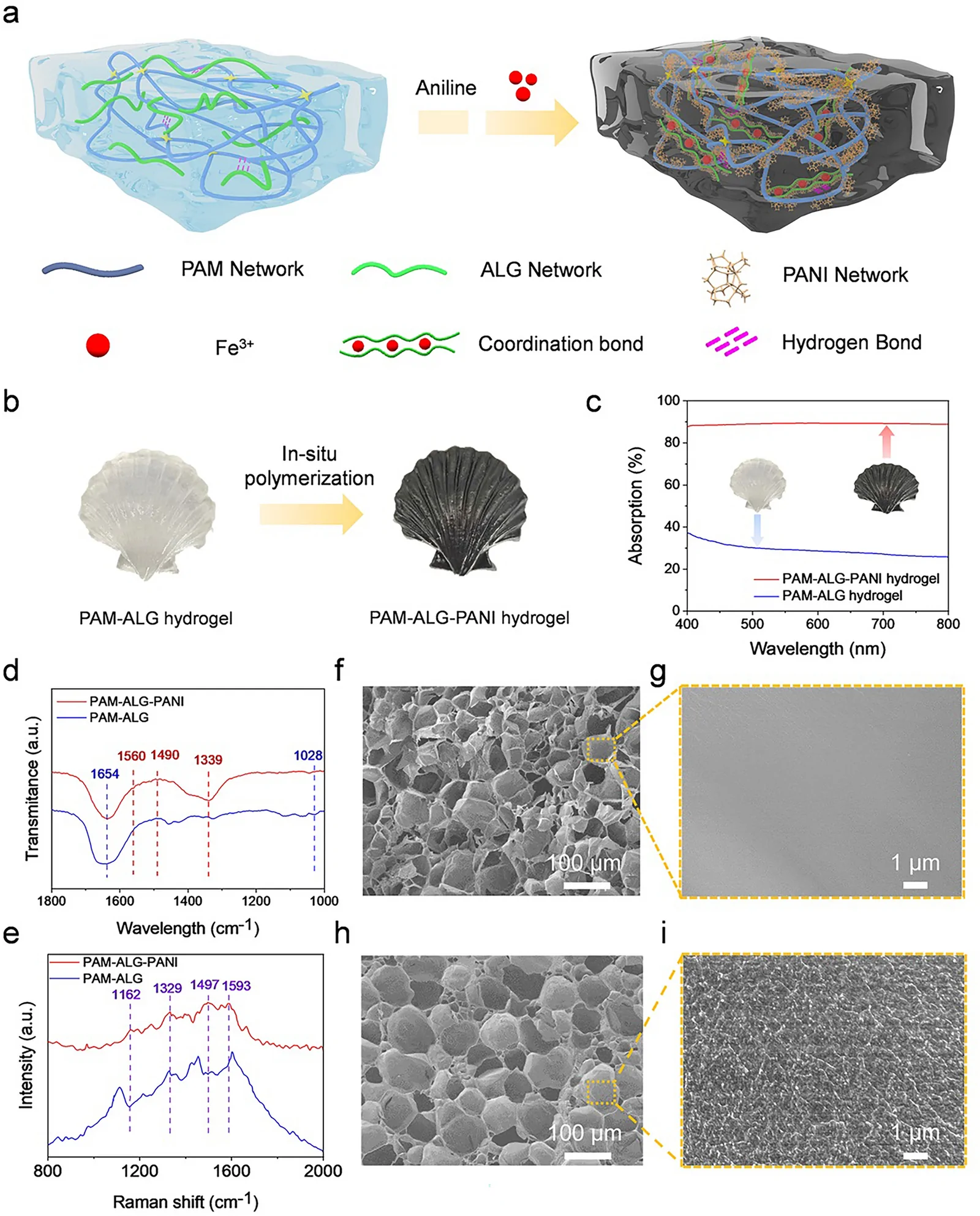
Preparation and characterization of black conductive polymer hydrogels. **a** Schematic illustration of the preparation of a PAM–ALG–PANI hydrogel containing a multi-network structure with hydrogen and coordination bonds. **b** Optical photographs of black PAM–ALG–PANI hydrogels synthesized by in situ initiating ANI polymerization in a PAM–ALG matrix. **c** UV–Vis absorption spectra of PAM–ALG hydrogels before and after in situ polymerization of PANI. **d** Fourier transform infrared spectra (FTIR) and **e** Raman spectra of PAM–ALG and PAM–ALG–PANI hydrogels. SEM micrographs of PAM–ALG hydrogels at **f** low-magnification and **g** high-magnification scales. Corresponding SEM images of PAM–ALG–PANI hydrogels at** h** low and **i** high magnification levels

Figure 2a illustrates the preparation of black CPHs, which have covalently cross-linked and ionically cross-linked networks, by employing the in situ polymerization and post-cross-linking strategy. First, the covalently cross-linked polyacrylamide–sodium alginate (PAM–ALG) semi-interpenetrating network hydrogel is placed in a solution containing aniline (ANI) and hydrochloric acid, enabling the ANI monomer to penetrate the hydrogel network. Subsequently, the treated PAM–ALG hydrogels are submerged in a solution with Fe^3+^ to initiate the in situ polymerization of polyaniline (PANI) and post-cross-linking of ALG, resulting in the production of black PAM–ALG–PANI CPHs. The in situ polymerized PANI introduces a conjugated molecular structure, which facilitates the retention and transfer of electrons within its molecular chain, thereby providing an enhanced electron transport pathway for hydrogels. Notably, the Fe^3^⁺ ion serves a dual role during in situ polymerization, acting as both an initiator for the PANI polymerization and a cross-linking agent. These trivalent cations establish coordination bonds with the imine group on the PANI chain and the carboxyl group on the ALG backbone, forming a strong ionic cross-linking network [38, 39]. Moreover, the PAM–ALG–PANI hydrogel network contains abundant hydrogen bonding, formed between the amide group on PAM, the carboxyl and hydroxyl groups on ALG, and the imino group on PANI [40, 41]. Non-covalent interactions, including hydrogen and coordination bonds, effectively enhance the compatibility between hydrophobic PANI and hydrophilic PAM–ALG, thereby greatly improving the stability of the formed hydrogels. Furthermore, these non-covalent bonds serve as energy dissipation centers during mechanical deformation, resulting in a strong load-bearing capacity. Therefore, the prepared PAM–ALG–PANI hydrogels possess mechanical robustness and enhanced electrical conductivity, showing significant potential for applications in flexible sensing and wearable electronics.

Benefiting from the plasticity and controllability of the hydrogel preparation process, hydrogel structures with different shapes and morphologies, such as conchs, starfish, shells, and leaves, can be precisely customized (Fig. S1). As shown in Fig. 2b, the transparent PAM–ALG hydrogel is transformed into a black PAM–ALG–PANI hydrogel after in situ polymerization of PANI in the hydrogel matrix. Notably, the prepared black PAM–ALG–PANI hydrogel exhibits high absorbance (absorption rate > 88%) throughout the visible range, which is much higher than that of the PAM–ALG hydrogel (Fig. 2c). This high absorbance endows the PAM–ALG–PANI hydrogel with the ability to absorb incoherent scattered light, acting as a structural color enhancement [32, 33]. The water content of the PAM–ALG hydrogel is 84.11%, which slightly increases to 87.58% after the in situ polymerization of PANI, and then gradually decreases with the increase in the ANI content (Fig. S2). To investigate the alteration in the chemical composition of the hydrogels before and after the reaction, the PAM–ALG and PAM–ALG–PANI hydrogels are analyzed using infrared spectroscopy and Raman spectroscopy. The characteristic peaks of PAM and ALG appear in the infrared spectrograms of both hydrogels, where the peak at 1654 cm^−1^ is attributed to the C = O stretching vibration of PAM [42], and the peak at 1028 cm^−1^ corresponds to the deformation vibration of the C–O–C or C–O–H moiety of ALG (Fig. 2d) [43]. This result indicates that the PAM–ALG primary hydrogel network was successfully synthesized by free radical polymerization. After introducing PANI, a series of new absorption peaks appear, where the peaks at 1560, 1490, and 1339 cm^−1^ are attributed to the deformation of the quinone ring and the vibration of the benzene ring, as well as the C–N stretching of the phenylenediamine, respectively [43, 44]. Similarly, a series of characteristic peaks of PANI are observed in the Raman spectra, including C = C stretching of the quinone rings at 1593 cm^−1^, C–H bending of the quinone rings at 1162 cm^−1^, and C–N·^+^ stretching at 1329 cm^−1^, further proving the successful formation of the PANI network (Fig. 2e) [45].

The micro-morphology of the hydrogels before and after in situ polymerization of PANI is observed by scanning electron microscopy (SEM). As shown in Fig. 2f, the PAM–ALG hydrogel shows a sponge-like porous architecture, which facilitates the adsorption of ANI monomers, allowing ANI to permeate the hydrogel network for polymerization. At higher magnification, the pore walls of the PAM–ALG hydrogel reveal a smooth surface morphology (Fig. 2g). After in situ polymerization of PANI, the PAM–ALG–PANI hydrogels still retain the three-dimensionally interconnected porous structure (Fig. 2h). Compared with the featureless surface of the pristine PAM–ALG, the high-resolution SEM reveals that the PAM–ALG–PANI exhibits a rough textured morphology (Fig. 2i). These microstructural modifications resulted from the uniform deposition of PANI aggregates along the polymer backbone of the hydrogel, confirming the successful establishment of the conductive network. The porous structure and three-dimensional interconnected conductive network of PAM–ALG–PANI hydrogels facilitate rapid electron transport, making them effective as conductive materials. The conductivity of PAM–ALG–PANI hydrogels shows a nonlinear relationship with the ANI monomer content (Fig. S3). With the increase in the ANI content from 1 to 4 wt.%, the hydrogel reaches a peak conductivity of 4.08 S m^−1^ at 3 wt.% of ANI content, beyond which the excessive aggregation of PANI disrupts the percolation network, resulting in a diminished conductivity. Additionally, the conductivity of the hydrogel increased slightly with increasing temperature (from 20 to 40 °C) and humidity (from 30% to 90%).

### Mechanical Properties of PAM–ALG–PANI Hydrogels

**Fig. 3:**
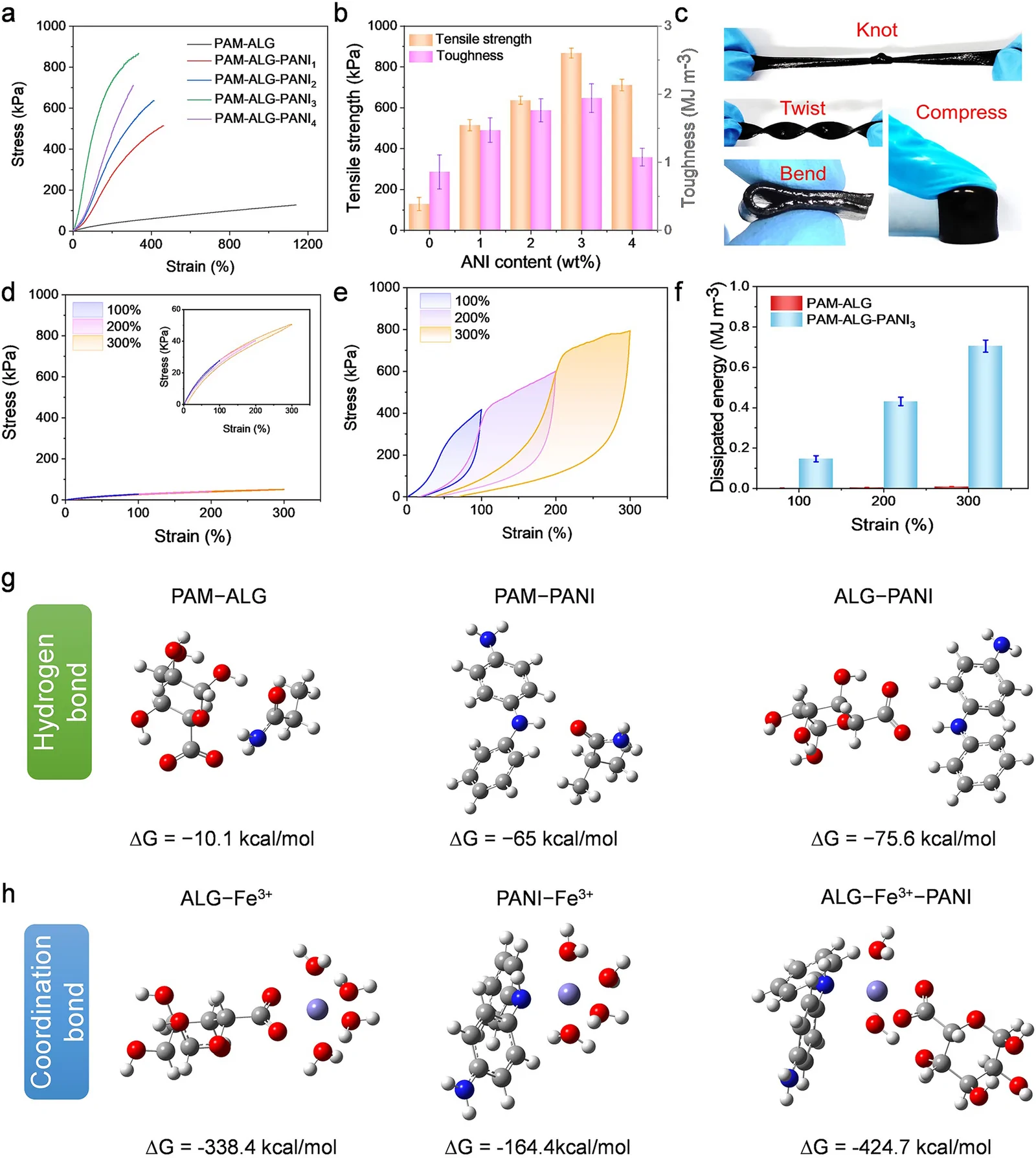
Mechanical performance of hydrogels. **a** Tensile stress–strain curves of hydrogels with varying ANI contents and **b** their corresponding tensile strength and toughness. **c** Optical images demonstrate the capability of PAM–ALG–PANI hydrogel to withstand different mechanical stresses. Loading–unloading curves of the **d** PAM–ALG and **e** PAM–ALG–PANI_3_ hydrogels with **f** energy dissipation calculated from hysteresis loop areas. DFT simulations of PAM–ALG–PANI hydrogel illustrating **g** hydrogen and** h** coordination bonds between polymer structural units

The mechanical properties of hydrogels are systematically investigated through uniaxial tensile and compression testing. Figure 3a presents representative tensile stress–strain curves for PAM–ALG–PANIx hydrogels with varying ANI concentrations (denoted as x). The primary PAM–ALG hydrogel exhibits characteristics typical of soft, highly ductile materials, demonstrating a tensile strength of 128.0 kPa with an elongation at break of 1140.0%. Notably, introducing the PANI network induces substantial mechanical reinforcement, where the tensile strength displays a non-monotonic dependence on ANI concentration from 1 to 4%. At 1 wt.% ANI loading, the tensile strength increases to 514.1 kPa, accompanied by a decrease in elongation to 463.5%. This phenomenon suggests that introducing rigid PANI enhances the load resistance of the hydrogel, but simultaneously restricts the sliding of the polymer chains. As the ANI content increases to 3 wt.%, the tensile strength of the hydrogel increases by a factor of 6.7 reaching a peak value of 867.1 kPa, corresponding to a strain of 335.9%. Figure S4 shows that the PAM–ALG–PANI_3_ hydrogel can withstand 300% tensile deformation without breaking. In addition, the PAM–ALG–PANI hydrogel demonstrates a simultaneous improvement in both Young’s modulus and toughness. Moreover, after introducing PANI, the toughness of the hydrogel is improved, increasing with the ANI content until reaching a turning point at 3 wt% (Fig. 3b). The initial toughness of the PAM–ALG hydrogel is 0.85 MJ m^−3^, while the toughness of the optimized PAM–ALG–PANI_3_ hydrogel increases to 1.94 MJ m^−3^. Analogous reinforcement behavior is observed in compression tests, revealing maximum compressive strength and modulus at 3 wt.% ANI content, followed by a gradual decline with higher loading. Under 80% strain, the compression strength increases 13 times from 0.131 to 1.75 MPa (Fig. S5), and the compression modulus increases from 37.6 kPa for PAM–ALG to 131.5 kPa for PAM–ALG–PANI_3_ (Fig. S6). As shown in Fig. 3c, PAM–ALG–PANI can withstand various mechanical stimuli, including knotting, twisting, bending, and compression, revealing its tolerance to complex deformations.

The energy dissipation behavior of hydrogels is evaluated by tensile loading–unloading tests. Figure 3d, e presents the loading–unloading curves of PAM–ALG hydrogel and PAM–ALG–PANI hydrogel under various tensile strains, respectively, where the hysteresis loop area serves as a quantitative indicator of their energy dissipation capacity. The loading and unloading curves of the PAM–ALG hydrogel nearly completely overlap, and the hysteresis loop is barely visible. In contrast, PAM–ALG–PANI demonstrates a significant hysteresis loop, which increases significantly with increasing strain. Quantitative results for the hysteresis loop area reveal that the PAM–ALG–PANI hydrogel dissipates a large amount of energy during load bearing, compared to the negligible amount of energy dissipated by PAM–ALG. The energy dissipation is 0.147 MJ m^−3^ at 100% strain, 0.431 MJ m^−3^ at 200% strain, and 0.725 MJ m^−3^ at 300% strain, respectively. Furthermore, cyclic loading–unloading tests reveal structural softening after the first cycle, evidenced by a marked reduction in the hysteresis loop and an elastic modulus ratio (E_2_/E_1_) of 36% (Fig. S7). Fatigue testing results reveal that the PAM–ALG–PANI hydrogel exhibits initial stress softening during the early stage of stretching, a behavior that becomes more evident with increasing cycle numbers (Fig. S8). After 5000 cycles of stretching and releasing at 30% strain, the stress level of the hydrogel remains relatively stable, with an attenuation rate of 12.8%. This is attributed to the elastic recovery of the covalently cross-linked network and the reversible breakage and reformation of dynamic bonds. When the number of cycles increases to 10,000, the stress attenuation rate rises to 27.6%, indicating irreversible changes and fatigue damage in the hydrogel’s network structure.

DFT calculations systematically elucidate the molecular mechanism of the enhanced mechanical properties in PAM–ALG–PANI hydrogels. Hydrogen bond calculations reveal that the three components of the PAM–ALG–PANI hydrogel can form hydrogen bonds with each other (Figs. 3g and S9). In the PAM–ALG system, amide groups (− CONH₂) on PAM chains establish hydrogen bonds with both carboxyl (− COOH) and hydroxyl (− OH) moieties of ALG, exhibiting binding energies of ΔG =  − 10.1 kcal mol^−1^. In the PAM and PANI systems, the amide group (− CONH₂) of PAM forms hydrogen bonds with the amino group (− NH −) of PANI with a binding energy of ΔG =  − 65 kcal mol^−1^. In addition, the carboxyl group (− COOH) of ALG and the amine group (− NH −) of PANI can form hydrogen bonds with a higher binding energy (ΔG =  − 75.6 kcal mol^−1^). Through the dynamic combination of hydrophilic groups and polar groups of PANI chains, these hydrogen bond networks effectively overcome the interfacial incompatibility between hydrophobic PANI and hydrophilic PAM–ALG networks, thereby optimizing the stability of the multi-network structure. Furthermore, these hydrogen bonds can undergo dynamic fracture and reconstruction under external forces to assist the hydrogel in dissipating energy.

DFT further simulates the coordination bonds formed between the hydrated iron ions and ALG and PANI in the PAM–ALG–PANI hydrogel network (Figs. 3h and S10). The calculation results confirm that the carboxylate anion (− COO⁻) of ALG forms a strong metal coordination with Fe^3+^ (ΔG =  − 338.4 kcal mol^−1^), while the amine group (− NH −) of PANI can also interact with Fe^3+^ (ΔG =  − 164.4 kcal mol^−1^). Remarkably, the multidentate coordination involving both ALG’s carboxylate and PANI’s amine groups dramatically enhances the binding energy to ΔG =  − 424.7 kcal mol^−1^, indicating Fe^3^⁺ ions serve as robust dynamic cross-linkers bridging ALG and PANI domains. Notably, the binding energy of the coordination bond is significantly higher than the hydrogen bond, indicating that the coordination bonds play a dominant role in energy dissipation. The simulated mechanism was further verified by comparing the mechanical properties of PAM–ALG–PANI hydrogels prepared using APS as an initiator. The APS-initiated system with only hydrogen bonds, exhibits significantly inferior tensile strength (318.29 kPa) and toughness (0.26 MJ m^−3^) compared to the system with coordination bonds (867.1 kPa and 1.94 MJ m^−3^) (Fig. S11). Additionally, rheological measurements under alternating shear strain levels reveal a recoverable modulus change, confirming the existence of dynamic bonds within the PAM–ALG–PANI hydrogel network (Fig. S12). Therefore, under mechanical loading, hydrogen bonds play a supplementary role in energy dissipation through dynamic dissociation and reconfiguration, which facilitates low energy dissipation and localized stress redistribution. The macroscopic toughness is dominated by coordination bonds with strong binding energy, which absorb a large amount of energy through fracture during larger deformations, thereby allowing the hydrogel to maintain structural integrity under complex mechanical stress.

### Strain Sensing Properties of PAM–ALG–PANI Hydrogels

**Fig. 4:**
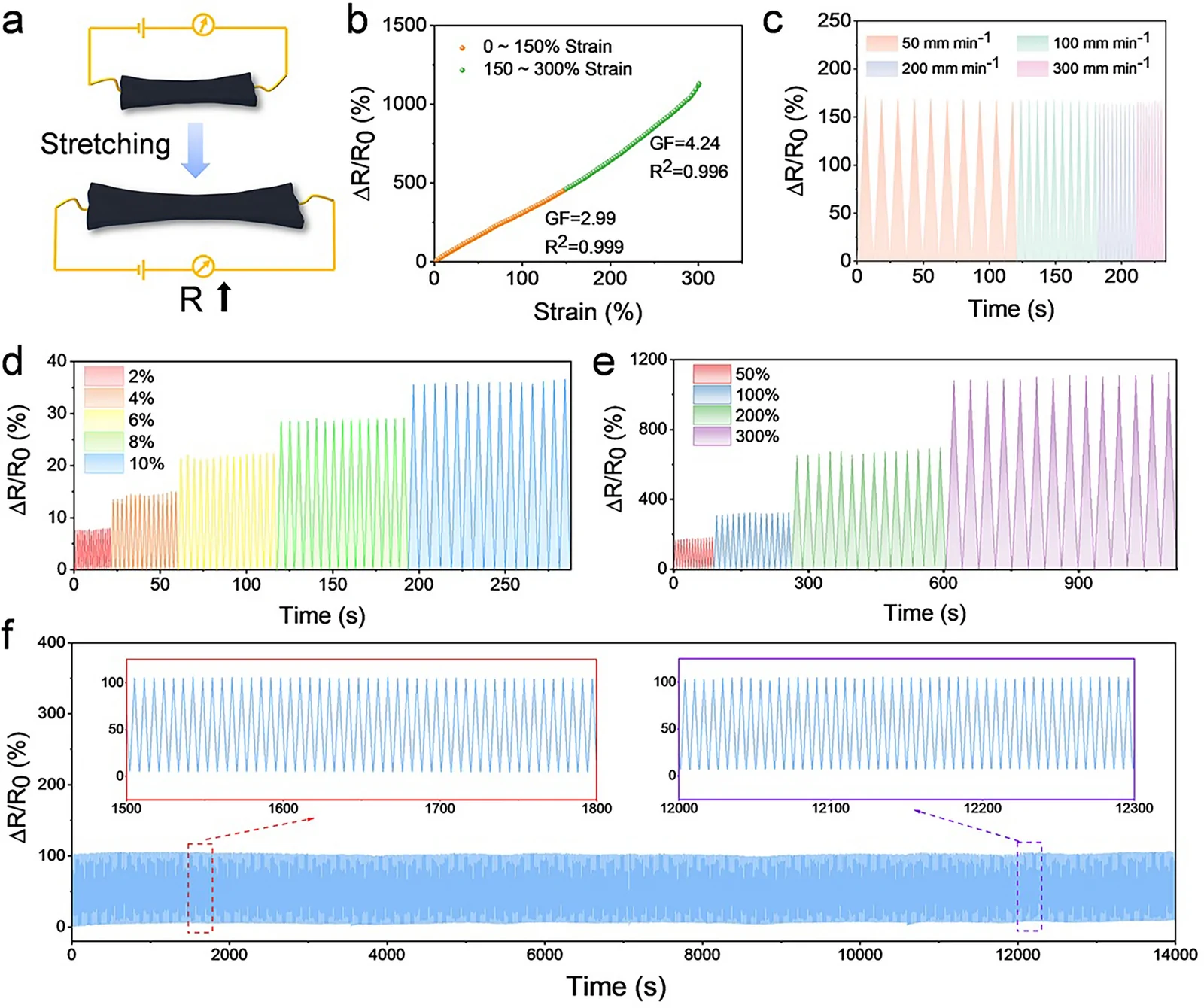
Strain sensing performance of the PAM–ALG–PANI hydrogels. **a** Sensing mechanism of hydrogel strain sensors. **b** Relative resistance variation and corresponding fitted curves over the strain range of 0 − 300%. **c** Relative resistance variations during cyclic loading–unloading at different speeds with a maximum strain of 50%. **d** Relative resistance variations at low strain (2%− 10%). **e** Relative resistance variations under high strains (50% − 300%). **f** Relative resistance variations for 2200 cycles of loading–unloading at 30% strain and 300 mm min^−1^ (Room temperature, RH = 72%). The inset is a magnified view of the real-time resistance signal within different stretching cycles

To evaluate the strain sensing properties of hydrogels, PAM–ALG–PANI hydrogels are assembled into resistive sensors as sensing elements (Fig. 4a). When the hydrogel is stretched, its sensing structure is geometrically deformed by the external force, resulting in a change in resistance to achieve the sensing function. As shown in Figs. 4b and S13, the relative resistance change curves of the hydrogel sensors display a trend of segmented linear growth with different slopes as the strain increases. The slope of the curve is defined as the gauge factor (GF), which is typically employed to evaluate the sensitivity of the sensor [44]. For the PAM–ALG–PANI hydrogel, the GF value is 2.99 at lower strains from 0 to 150%, and increases to 4.24 at higher strains from 150% to 300%. This nonlinearity arises from the strain-dependent changes in the contact behavior of conductive fillers. In the low-strain stage (0–150%), the conductive PANI fillers within the hydrogel network maintain stable contact, forming continuous interconnected conductive paths. This stable configuration results in a relatively small resistance change rate, hence the lower GF. As strain enters the higher range (150%–300%), further deformation induces sliding between adjacent conductive fillers, leading to a significant reduction in their contact area. This reduction in contact area causes the number of conductive paths to start decreasing, with some even beginning to disconnect partially. These changes enhance the sensitivity of resistance variation to strain, thereby increasing the GF [26]. In addition, the hydrogel sensor clearly monitors consistent and repetitive real-time electrical signals at both slow and fast loading–unloading rates, demonstrating its ability to respond and recover quickly in dynamic stress detection (Fig. 4c). Taking advantage of its high sensitivity, the hydrogel sensor can accurately capture the difference in resistance signals over a wide strain window, including small strains of 2% − 10%, and large strains of 50%− 300% (Fig. 4d, e). Under stepwise loading and unloading over a strain range of 0 − 100%, the sensor captured a stepwise electrical signal without any hysteresis or attenuation (Fig. S14). When the hydrogel sensor is subjected to 2200 loading and unloading cycles, the real-time resistance signal displays a constant and repeatable peak shape, demonstrating the excellent stability and reliability of PAM–ALG–PANI hydrogel for long-term sensing and monitoring (Fig. 4f). After being placed in a humid indoor environment for one week, the hydrogel sensor maintains stable electrical output through 2200 stretching cycles. Its relative resistance changes closely match that of fresh devices with negligible signal drift, confirming excellent long-term durability and environmental stability (Fig. S15). The LEDs connected to the hydrogel in the same conductive loop experience reversible changes in light and dark brightness during repeated stretching of the hydrogel, which further confirms the stability of the PAM–ALG–PANI hydrogel (Fig. S16).

### Integration and Characterization of DM-BCESC Sensors

**Fig. 5:**
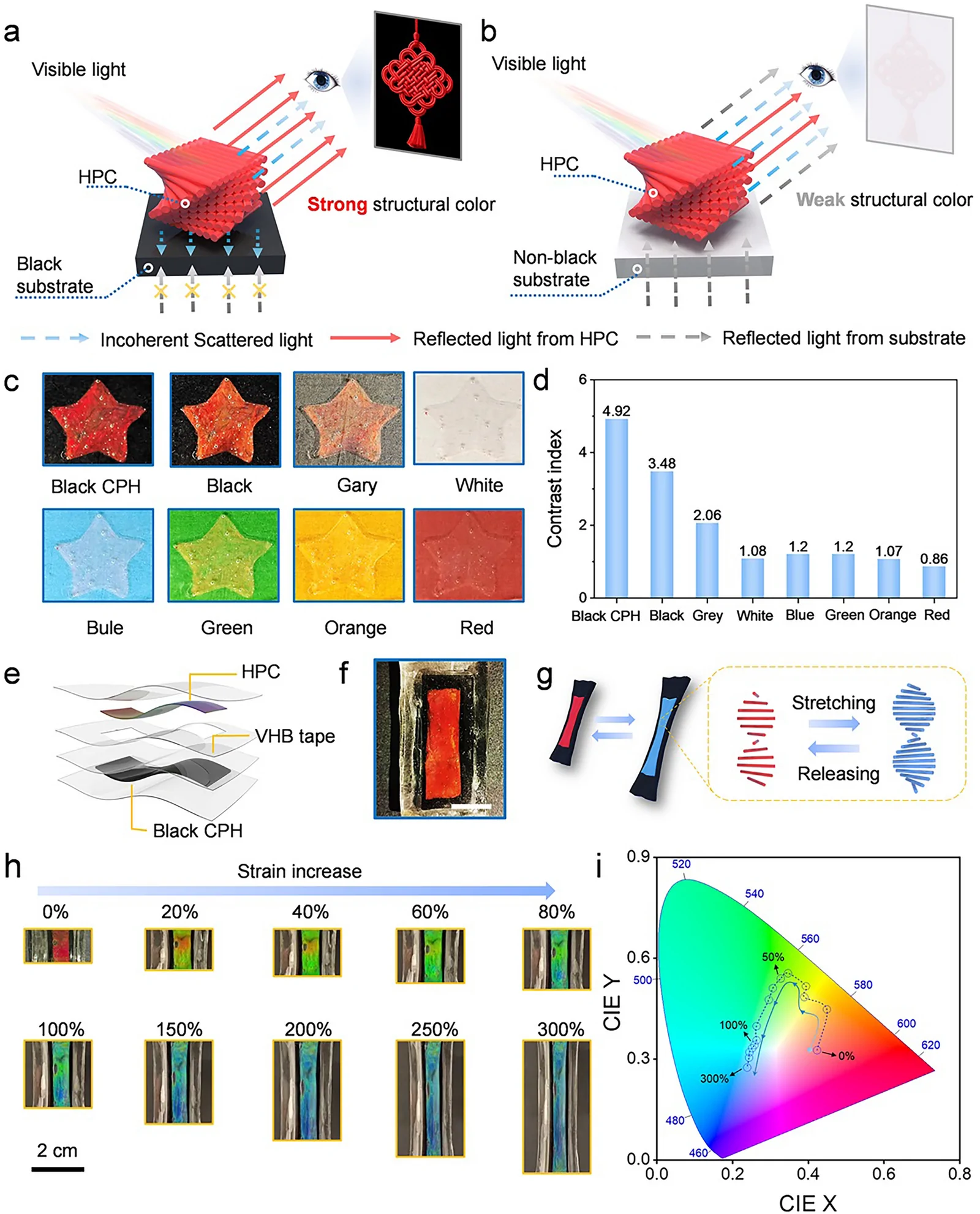
Integration and mechanochromic characterization of DM-BCESC sensors. **a** Mechanism diagram for structural color enhancement by a black substrate, and** b** structural color deterioration with a non-black substrate. **c** Photographs of structural color patterns in composite devices with substrates of different colors. **d** Contrast index of structural colors for composite devices with various substrate colors. **e** Schematic of the integration of the DM-BCESC sensor. **f** Photograph of the integrated DM-BCESC sensor (scale bar = 1 cm). **g** Schematic showing color and crystal structural alterations in the sensor’s HPC layer under tensile deformation. **h** Optical images of the sensor’s color change at different strain levels. **i** CIE chromaticity trajectory mapping illustrates strain-induced chromaticity transitions within the strain range of 0–300%

To develop multifunctional sensors integrating strain sensing and mechanochromic capabilities, we designed a multilayer device combining HPC with a stretchable conductive PAM–ALG–PANI hydrogel. In this device, the upper HPC layer self-assembles into a cholesteric liquid crystal, which generates structural color through Bragg reflection of visible light. Polarizing microscopy reveals that the assembled HPC exhibits distinct birefringence and characteristic fingerprint textures (Fig. S17). The underlying PAM–ALG–PANI black hydrogel serves not only as a sensing element, but also as a light-absorbing substrate to enhance the structural color display by absorbing incoherent scattered light. Figure 5a presents an interfacial light propagation model elucidating the color enhancement mechanism of the black substrate. When visible light strikes the device, the black substrate effectively absorbs the incoherent scattered light that penetrates the HPC layer. Moreover, the black substrate effectively mitigates spectral superposition interference from the substrate’s intrinsic coloration, thereby enhancing structural color saturation. As a result, the human eye mainly receives the reflected light generated by the HPC layer, thus observing a clear structural color pattern. On the contrary, devices featuring non-black substrates substantially diminish the visibility of structural color patterns. This occurs because strong incoherent scattering and the substrate’s inherent reflected light interferes with the optical signals of HPC, thereby preventing clear visual perception of the structural colors (Fig. 5b). This substrate-dependent color difference is visually demonstrated in Fig. 5c. The red HPC star pattern on black hydrogel substrates exhibits sharp boundaries and saturated coloration, whereas only faint structural color outlines with blurred edges are discernible on gray or colored substrates. Reflectance spectral analysis further confirms the critical role of substrate color. The composite system of HPC and black PAM–ALG–PANI hydrogel exhibits a single structural color reflection peak at 678 nm, aligning spectrally with pure black substrates (Fig. S18). Conversely, systems with gray/colored substrates show additional reflection peaks and baseline shifts alongside structural color signals. These extraneous peaks, observed in the reflectance spectra of pure substrates, originate from the substrates’ intrinsic reflection properties, indicating their interference with structural color signal extraction (Fig. S19).

A contrast index (CI) is adopted to quantify color performance, defined as the ratio of structural color to background substrate reflectance at 678 nm (the peak wavelength of maximum structural color). As quantified in Fig. 5d, the correlation between calculated CI values and digital images reveals that higher CI values correspond to more pronounced structural colors. The black hydrogel system with low reflectance achieves an ultra-high CI value of 4.92. This represents a 2.3 times improvement over the gray background (CI = 2.06) and up to 5.7 times enhancement relative to colored substrates (e.g., red, CI = 0.86). This result confirms that the black substrate effectively eliminates spectral interference from substrate color, improves the contrast of visual perception, thereby maximizing the structural color characteristics. Furthermore, absorption spectroscopy analysis indicates that the black hydrogel maintains excellent broadband absorption (> 88%) across the 400 − 800 nm wavelength range, comparable to pure black substrates while significantly outperforming non-black substrates (Fig. S20). This efficient light absorption capability captures incoherent scattered light passing through the HPC layer, reduces incoherent scattering interference, and enhances structural color observation. Taken together, the black hydrogel enhances HPC structural coloration through a dual mechanism by absorbing incoherent scattered light to minimize optical interference, and suppressing inherent substrate reflections to amplify color purity, thereby optimizing both optical contrast and signal specificity.

Furthermore, we fabricated a flexible sensor through assembly of HPC and PAM–ALG–PANI hydrogel by using transparent 3 M’s Very High Bond (VHB) tape as the bonding medium (Fig. 5e). The integrated sensor exhibits a multilayer architecture comprising a sensing layer of black hydrogel at the base and a vivid red structural color layer of HPC on the upper surface, which are bonded and encapsulated through VHB interlayers (Fig. 5f). Particularly, customized structural color patterning on the device surface can be achieved by modulating the geometric configuration of the intermediate VHB layer (Fig. S21). This sensor demonstrates excellent mechanical robustness and mechanochromic performance, sustaining various deformations including stretching, bending, and 350 g weight loading, all accompanied by color responses (Fig. S22). Moreover, under prolonged and repeated bending stress, the VHB encapsulation layer maintains strong interfacial adhesion, ensuring the long-term integrity of the packaged structure (Fig. S23).

The mechanochromic mechanism originates from compressive deformation of the cholesteric helical structure in HPC under tension, leading to blue shift in Bragg reflection wavelength (Fig. 5g). As illustrated in Figs. 5h and S24, progressive tensile deformation triggers continuous color evolution in the HPC layer, with structural coloration transitioning through red–green–blue spectra as strain increases from 0 to 300%. CIE chromaticity analysis reveals that within the 0 − 100% strain range, a significant shift in chromaticity occurs with every 20% strain increment, spanning the red, green, and blue color gamuts (Fig. 5i). When strain exceeds the critical value of 100%, chromaticity coordinates exhibit only slight shifts within the blue region.

To quantitatively model the continuous relationship across the full strain range and elucidate the correlation between optical and electrical signals, Fig. S25 presents fitted functional relationships between strain and both CIE chromaticity coordinates and relative resistance change. Within the 0–100% critical strain interval, pronounced chromatic shifts occur in strict synchrony with linear resistance increases, enabling real-time ternary mapping of strain, color, and resistance. Beyond 100% strain, chromaticity coordinates stabilize, forming a distinct visual plateau. In contrast, the rate of resistance change surges, effectively compensating for diminished sensitivity in the color signal. This dynamic interplay establishes the color plateau as an intuitive visual threshold indicator for strain overload, while resistance provides continuous, precise quantification throughout the strain spectrum.

### DM-BCESC Sensors for Rehabilitation Training Monitoring

**Fig. 6:**
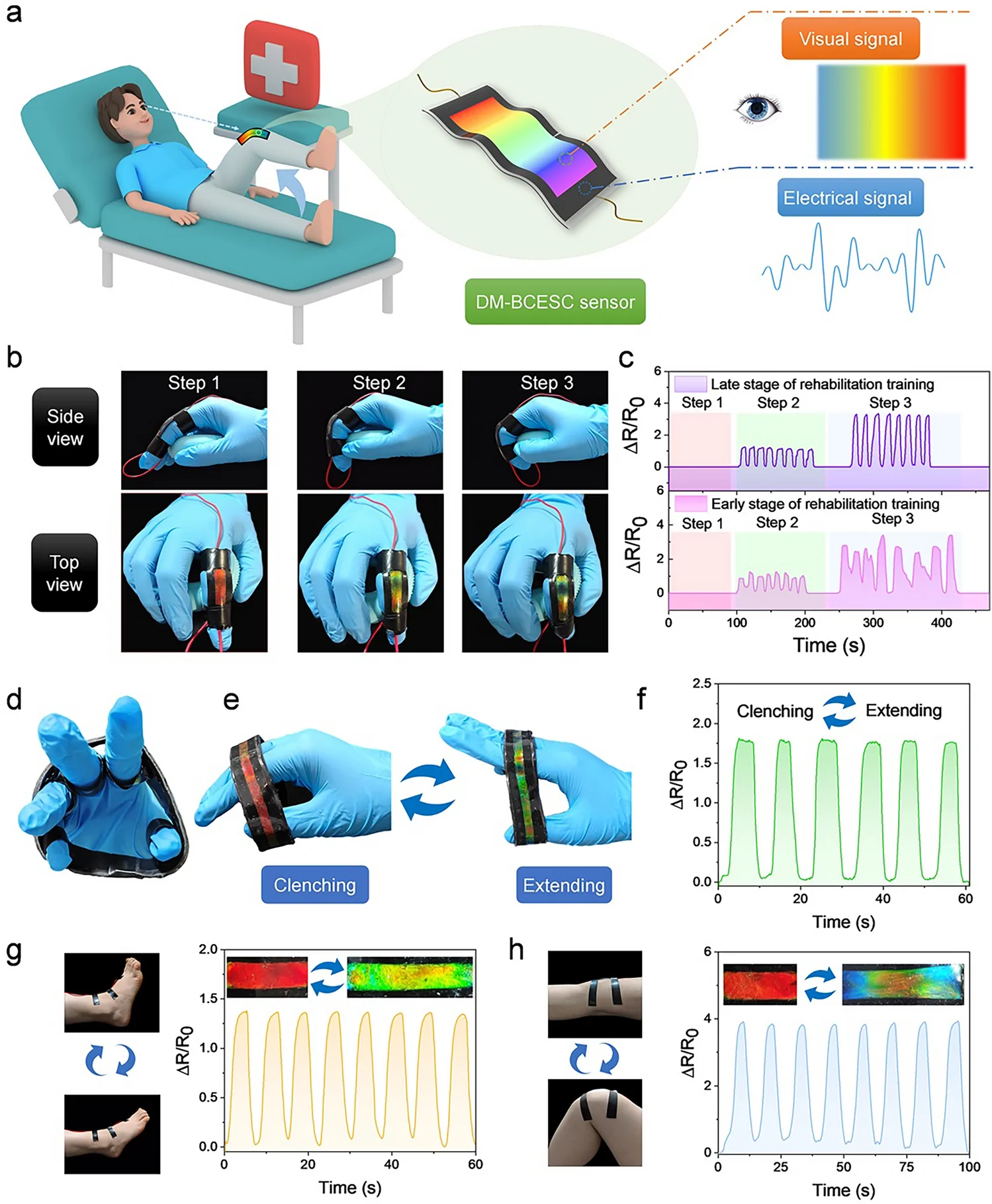
Applications of the DM-BCESC sensor in rehabilitation training monitoring. **a** Schematic diagram illustrating the operational mechanism of the DM-BCESC sensor. **b** Side and top views of the sensor during distinct stages of grip strength ring training, with **c** associated relative resistance change curves for each stage. **d** Image of the wearable finger elastic band integrated with DM-BCESC sensors. **e** Photograph depicting color changes in the wearable finger elastic band during finger flexion–extension training. **f** Resistance signals synchronously captured by the sensor during finger flexion–extension movements. **g** Simultaneous color transitions and electrical response behavior recorded by sensors during ankle movements. **h** Color variations and resistance signals monitored by the sensor during knee joint rehabilitation training

Stroke patients often experience limb motor dysfunction, including compromised finger flexion/extension and reduced wrist/ankle joint mobility, necessitating precise assessment methods to develop personalized rehabilitation programs for patients [46, 47]. To address this problem, we integrated the PAM–ALG–PANI hydrogel with exceptional electromechanical properties and the HPC with enhanced structural color, developing a DM-BCESC sensor for assessing rehabilitation training (Fig. 6a). This sensor is capable of synchronously capturing resistance changes and structural color variations induced by limb movements during rehabilitation training, creating a dual feedback mechanism that combines digitization and visualization. A comparison of the dual-mode sensor’s performance with other previously reported works is provided in Fig. S26. The designed dual-mode sensor demonstrates significant advantages in background interference resistance, sensitivity, color saturation, tensile strength, and cycle number. Furthermore, the DM-BCESC sensor demonstrates robust environmental stability, consistently delivering stable optical and electrical signal outputs unaffected by varying humidity levels (Fig. S27). The sensor also maintains robust performance and stable signal output during actual wear, even when exposed to near-body temperature and sweat for extended periods, proving its suitability for practical wearable applications (Fig. S28). Before evaluating its rehabilitation performance, a biocompatibility assessment confirmed the sensor’s biocompatibility, showing no observable cytotoxicity and supporting its suitability for on-body studies (Fig. S29).

To validate the DM-BCESC sensor’s effectiveness in rehabilitation assessment, this study employed volunteers to mimic limb motor dysfunction in stroke patients. Grip strength rings serve as rehabilitation training devices designed to restore finger muscle strength in patients. When volunteers perform finger grip strength training, as the range of finger joint flexion increases (the joint bending angle decreases), the sensors mounted on the fingers register a continuous color transition from red through green to blue (Figs. 6b and S30). This dynamic color gradient provides trainees with real-time visual feedback, significantly enhancing the engagement and initiative in rehabilitation training. Additionally, the electrical signals captured by the sensors exhibit a continuous increase in intensity as the degree of finger flexion increases, which clearly reflect the amplitude and frequency parameters of the movements in different training phases (Fig. 6c). Notably, comparative analysis of monitoring data across various rehabilitation stages reveals distinct signal patterns, with the initial phase characterized by irregular electrical fluctuations and the advanced phase showing stable, periodic signal characteristics. Therefore, the electrical signals acquired by the sensor provide a robust quantitative foundation for digital rehabilitation assessment, facilitating accurate tracking of recovery progression.

Bland–Altman analysis demonstrated strong agreement between joint flexion angles measured by the dual-mode sensor and the actual angles, confirming a reliable correlation between color variation and true joint flexion (Fig. S31). Conversely, measurements from the single-mode resistive sensor showed significant deviations from actual angles and greater data variability (Fig. S32). This discrepancy stems from the single-mode sensor’s reliance solely on electrical signal feedback, which lacks an intuitive visual mechanism and requires external devices for signal interpretation, resulting in noticeable judgment delays during training. Critically, the dual-mode sensor integrates visual feedback while maintaining precision in electrical signal measurement. As finger bending angles change during training, the sensor produces clearly distinguishable color and electrical signals (Fig. S33). This integrated feedback prompts patients to adjust their movements, thereby improving training compliance and movement accuracy.

The DM-BCESC sensor is innovatively designed as a wearable finger elastic band to monitor real-time motor function recovery during finger flexion–extension training (Fig. 6d). When the subject wears the device for rehabilitation, the sensor exhibits a reversible structural color transition from red to green corresponding to finger movements from flexion to extension, providing intuitive visual feedback on joint range of motion (Fig. 6e). Simultaneously acquired electrical signals demonstrate highly consistent response characteristics during repetitive cyclic testing, confirming excellent electromechanical coupling stability (Fig. 6f). Additionally, to validate the sensor’s versatility in rehabilitation monitoring, we extend its application to lower limb joint assessment. During ankle dorsiflexion training, the sensor displays red–green color transitions while generating electrical signals with amplitude-dependent regularity that precisely match movement ranges (Fig. 6g). For high-curvature knee bending movements, the sensor exhibits exceptional deformation adaptability, with its structural color progressively transitioning from red to blue as bending increased. The electrical signal module synchronously captures sinusoid-like waveforms, where the peak–valley difference corresponds to the knee flexion angle (Fig. 6h). In conclusion, such DM-BCESC sensing platform achieves not only quantitative analysis of multi-joint kinematic parameters but also establishes real-time visual feedback through intuitive color mapping. The synchronized chromatic–electrical response mechanism provides an innovative solution for smart rehabilitation assessment, demonstrating significant potential in wearable medical monitoring and digitized evaluation of motor function recovery.

## Conclusion

We have demonstrated a DM-BCESC sensor that integrates strain sensing and a visual interaction interface by utilizing black CPH substrates to enhance HPC structural color. This design is inspired by the phenomenon of melanin-enhanced structural color in Cyanocitta stelleri’s feathers. The DM-BCESC sensor comprises two core components, including a sensing substrate made of PAM–ALG–PANI CPH and a visual interface constructed from HPC liquid crystals. This HPC-based visual interface enables real-time conversion of mechanical stimuli into iridescent structural color variations. Significantly, the black PAM–ALG–PANI hydrogel plays a crucial role in enhancing the visibility of HPC structural color. Comparative analysis reveals that HPC on non-black substrates exhibits barely perceptible coloration, while HPC on black hydrogel substrates produces vivid structural colors readily discernible to the naked eye. This enhancement arises because the highly light-absorbing black hydrogel effectively absorbs incoherent scattered light and eliminates background light noise, thereby significantly improving the saturation of structural color. Additionally, by introducing hydrogen bonds and coordination bonds into the hydrogel network, PAM–ALG–PANI exhibits excellent electromechanical properties, ensuring stable and reliable strain monitoring. In stroke rehabilitation monitoring applications, the integrated DM-BCESC system provides synergistic feedback through concurrent electrical signal detection and visual interaction. When joint movement induces deformation, the visual interface immediately displays distinct color changes, assisting trainees in correcting their movements and boosting motivation. Concurrently, the CPH substrate synchronously outputs precise electrical signal parameters, including motion amplitude and frequency. This dual-mode sensing platform combines dynamic visual interaction for user engagement with quantitative digital assessment capabilities, demonstrating promising potential in rehabilitation medicine, human–machine interfaces, and smart wearable devices.

## Supplementary Information

Below is the link to the electronic supplementary material.

Supplementary file1 (DOCX 27464 KB)
