# Supplementary material for: Dual-Mode Sensor with Saturated Mechanochromic Structural Color Enhanced by Black Conductive Hydrogel for Interactive Rehabilitation Monitoring
Source: Nanomicro Lett. 2026 Jan 11;18:110. doi: 10.1007/s40820-025-01963-2 (PMC12790551; doi:10.1007/s40820-025-01963-2)
Supplement: Supplementary file 1 — Supplementary file1 (DOCX 27464 KB) [file 40820_2025_1963_MOESM1_ESM.docx]

Supporting Information for

**Dual-Mode Sensor with Saturated Mechanochromic Structural Color Enhanced by Black Conductive Hydrogel for Interactive Rehabilitation Monitoring**

Zhiyuan Sun^1,2, £^, Binhong Yu^1,2, £^, Chao Dong^3^, Chengjun Yu^1,2^, Lianghe Sheng^4^, Zhe Cui^1^, Yaming Liu^1,2^, Zhenni Lu^1,2^, Bingda Chen^5^, Daixi Xie^5^, Zhandong Huang^6^*, Songshan Zeng^1,2^*, Qingdong Ou^1,2^*

^1^ Macao Institute of Materials Science and Engineering (MIMSE), Faculty of Innovation Engineering, Macau University of Science and Technology, Taipa, Macao 999078, P. R. China

^2^ Macau University of Science and Technology Zhuhai MUST Science and Technology Research Institute, Zhuhai 519031, P. R. China

^3^ Chemistry and Physics Department, College of Art and Science, The University of Texas of Permian Basin, Odessa, TX 79762, United States

^4^ Department of Oncology, The First Affiliated Hospital of Jinan University, Guangzhou 510630, P. R. China

^5^ Key Laboratory of Green Printing, Institute of Chemistry, Chinese Academy of Sciences, Beijing Engineering Research Center of Nanomaterials for Green Printing Technology, Beijing National Laboratory for Molecular Sciences (BNLMS), Zhongguancun North First Street 2, 100190 Beijing, P. R. China

^6^ School of Chemical Engineering and Technology, Xi’an Jiaotong University, Xi’an 710049, P. R. China

^£^ Zhiyuan Sun and Binhong Yu contributed equally to this work.

*Corresponding authors. E-mail: [huangzhandong@xjtu.edu.cn](mailto:huangzhandong@xjtu.edu.cn) (Zhandong Huang); [sszeng@must.edu.mo](mailto:sszeng@must.edu.mo) (Songshan Zeng); [qdou@must.edu.mo](mailto:qdou@must.edu.mo) (Qingdong Ou)

**Supplementary Figures**


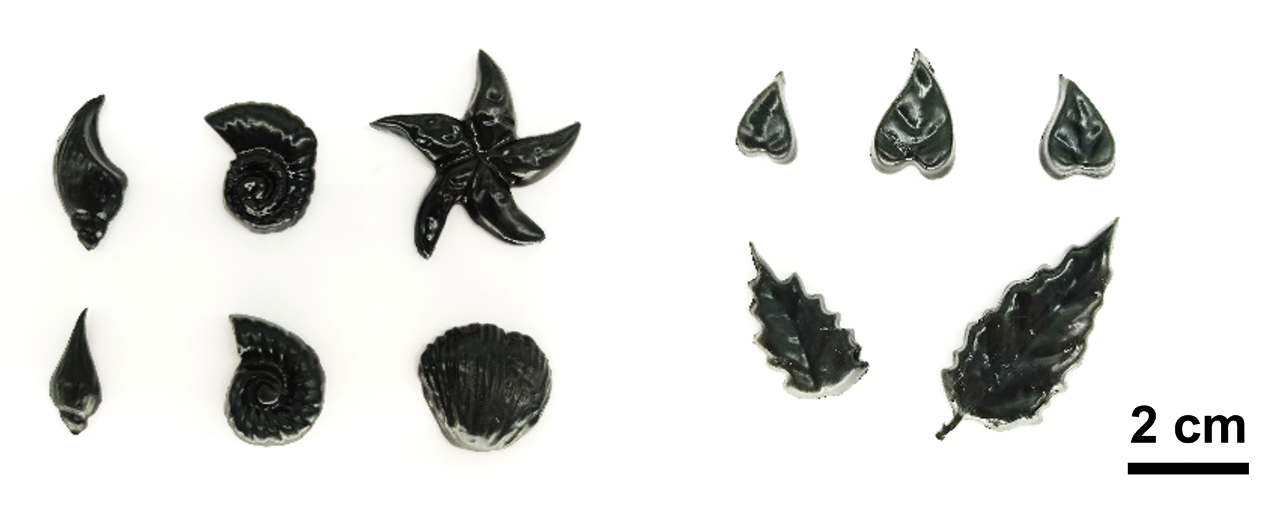


**Fig. S1** Photographs of PAM−ALG−PANI hydrogels with different patterns prepared by the molding method


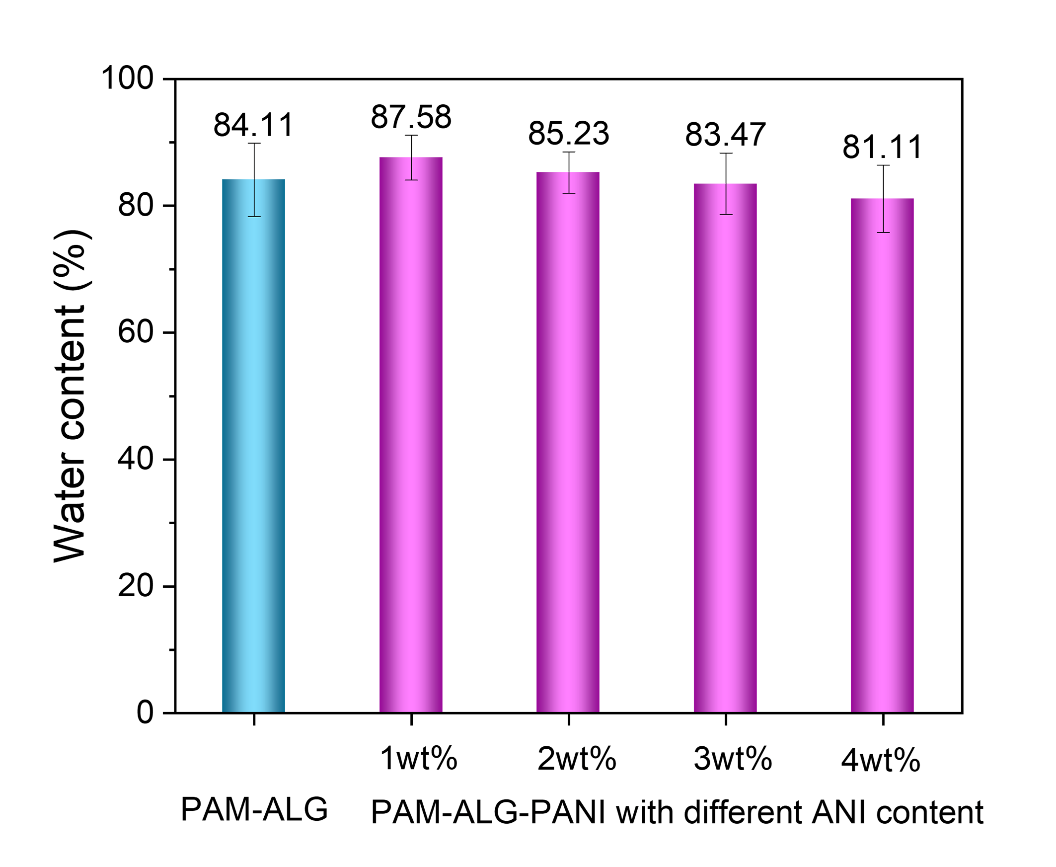


**Fig. S2** Water content of PAM−ALG and PAM−ALG−PANI hydrogels with different ANI contents


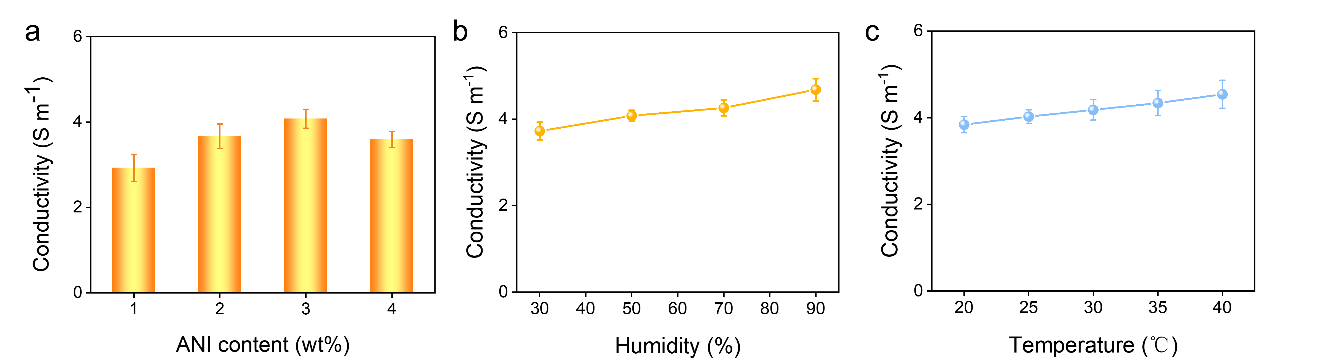


**Fig. S3** **a** Conductivity of PAM-ALG-PANI hydrogels with different ANI contents. **b** Conductivity of PAM-ALG-PANI3 hydrogel under various humidity conditions ranging from 3.72 to 4.68 S m^-1^. **c** Conductivity of PAM-ALG-PANI_3_ hydrogel at various temperatures ranging from 3.84 to 4.54 S m^-1^


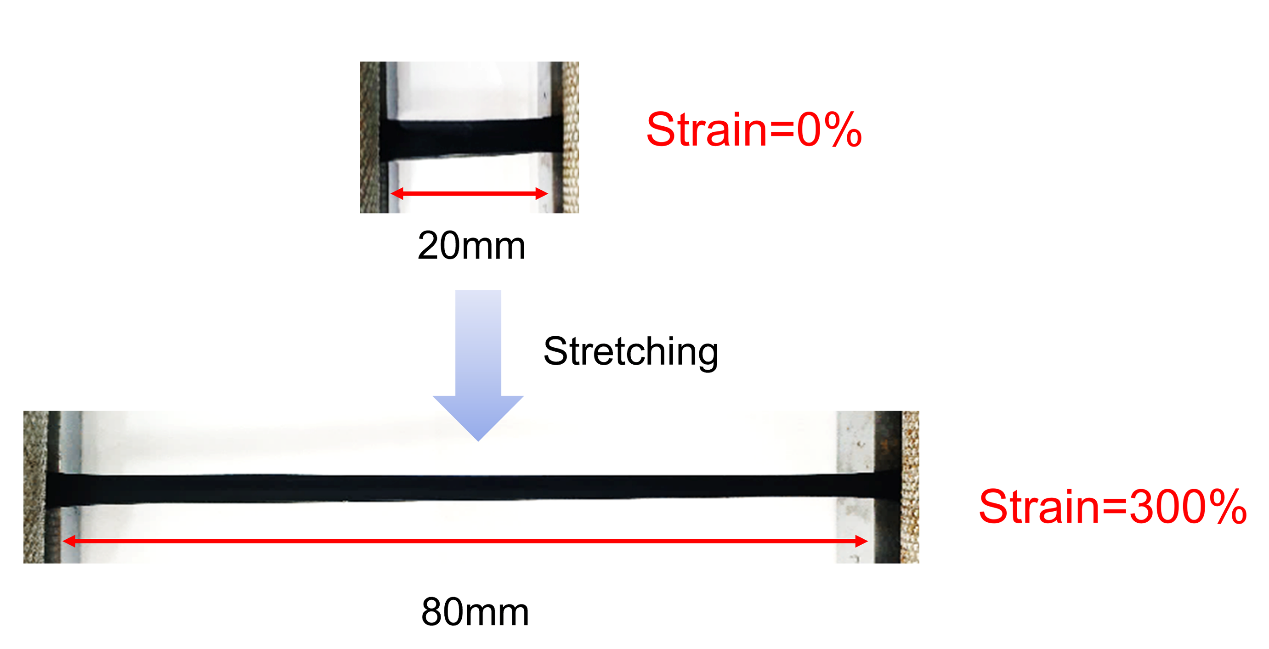


**Fig. S4** Photograph of PAM−ALG−PANI hydrogel stretched to a 300% strain


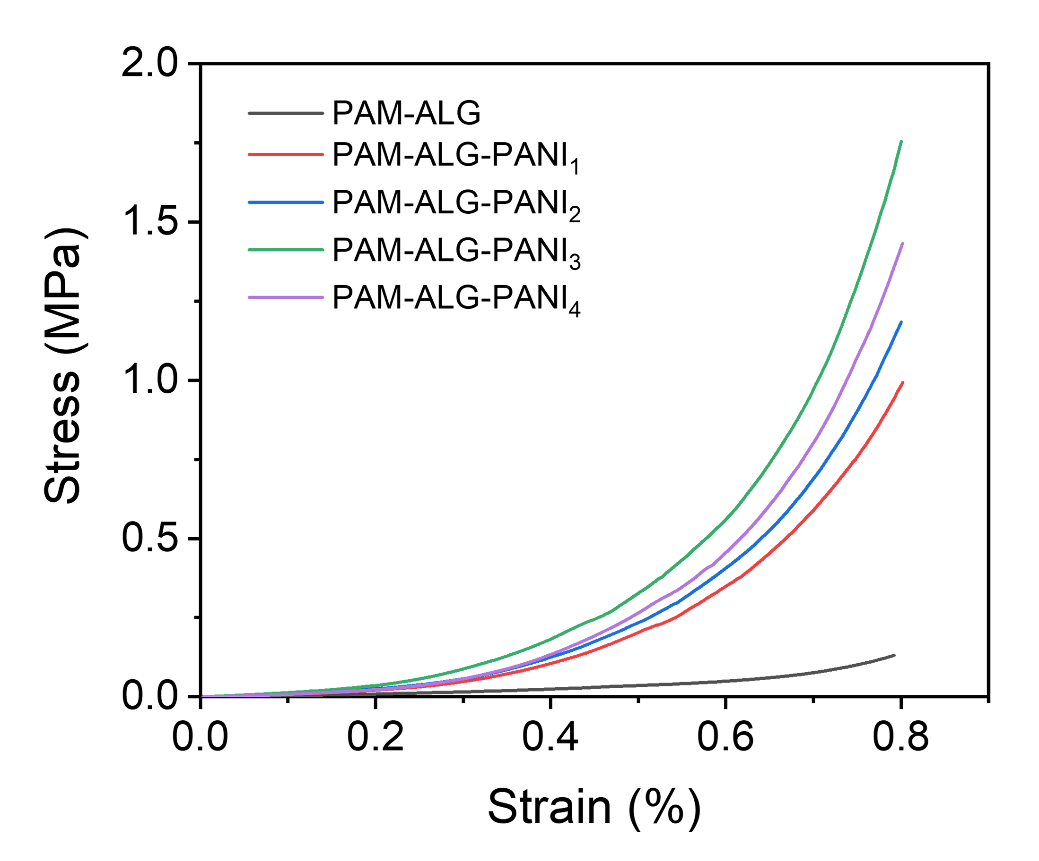


**Fig. S5** Compression performance of PAM−ALG−PANI hydrogels with different ANI contents


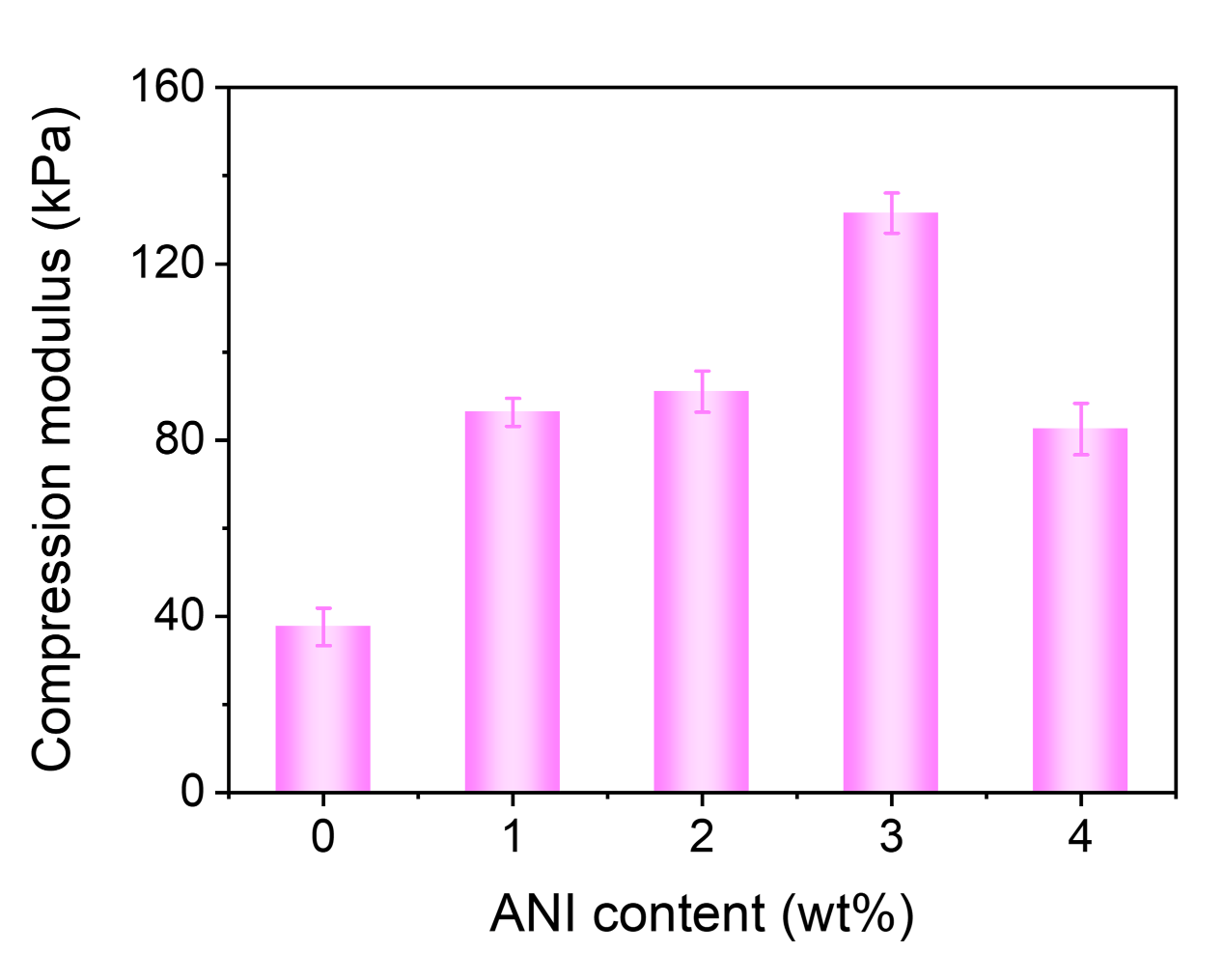


**Fig. S6** Compression modulus of PAM−ALG−PANI hydrogels with different ANI contents


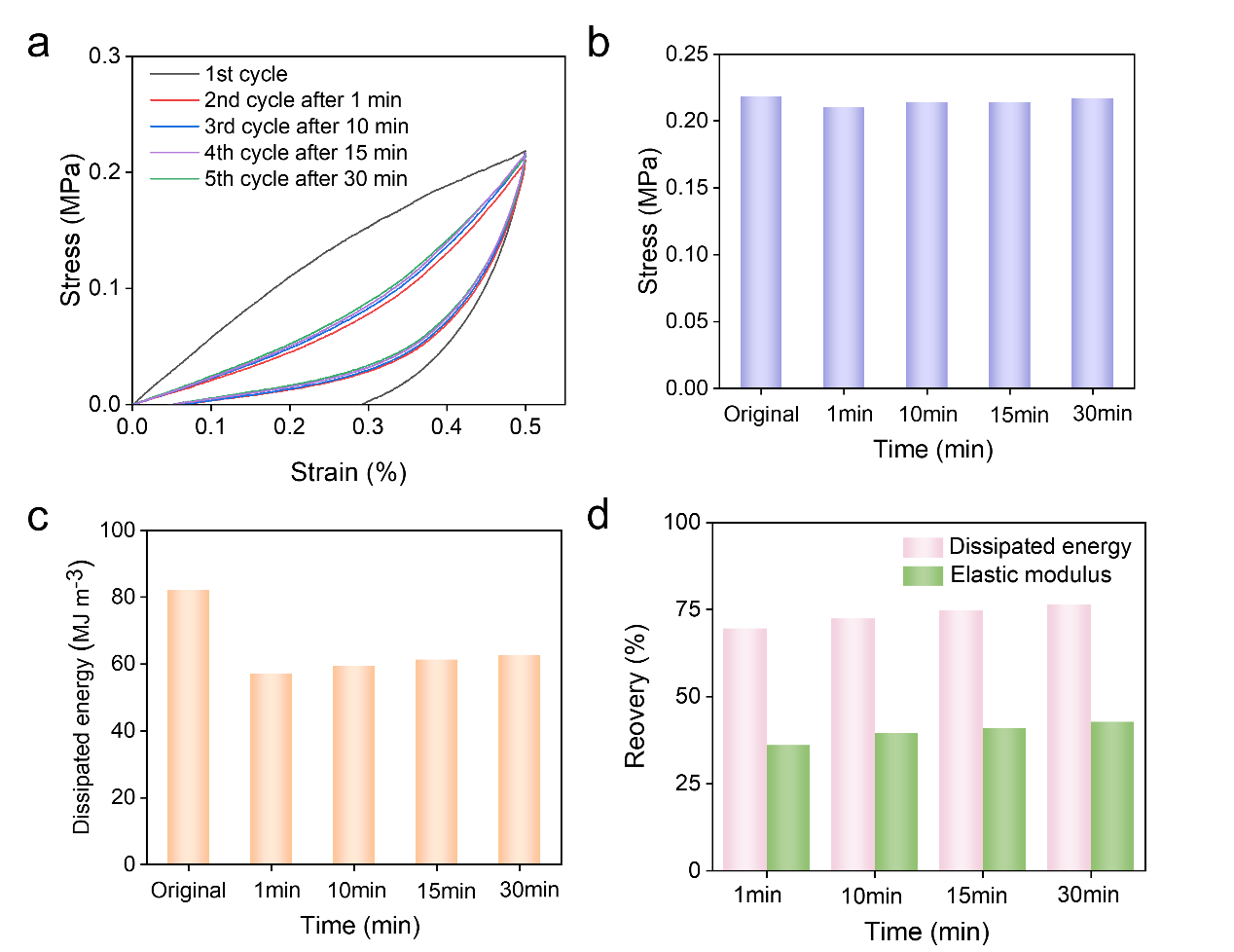


**Fig. S7 a** Five loading-unloading cycles of the hydrogel under 50% strain at different recovery times. **b** Strain variation of the hydrogel at different recovery times. **c** Dissipated energy variation of the hydrogel at different recovery times. **d** Energy dissipation recovery rate of the hydrogel. Cyclic loading-unloading tests are performed on PAM-ALG-PANI hydrogels. The results indicate a pronounced hysteresis loop in the first cycle, demonstrating a high energy dissipation capacity. In subsequent cycles, the hysteresis loop diminishes significantly, suggesting microstructural reorganization within the hydrogel network and noticeable softening. The ratio of the elastic modulus after the second loading to that after the first loading (E₂/E₁) is 36%. Furthermore, recovery behavior over time after initial loading is observed. The hysteresis loop is found to increase within 10 minutes and then nearly stabilize. The energy dissipation recovery rates and modulus efficiency measured at 1, 5, 15, and 30 minutes reach 69.5%/36%, 72.4%/39.5%, 74.75%/40.9%, and 76.3%/42.7%, respectively


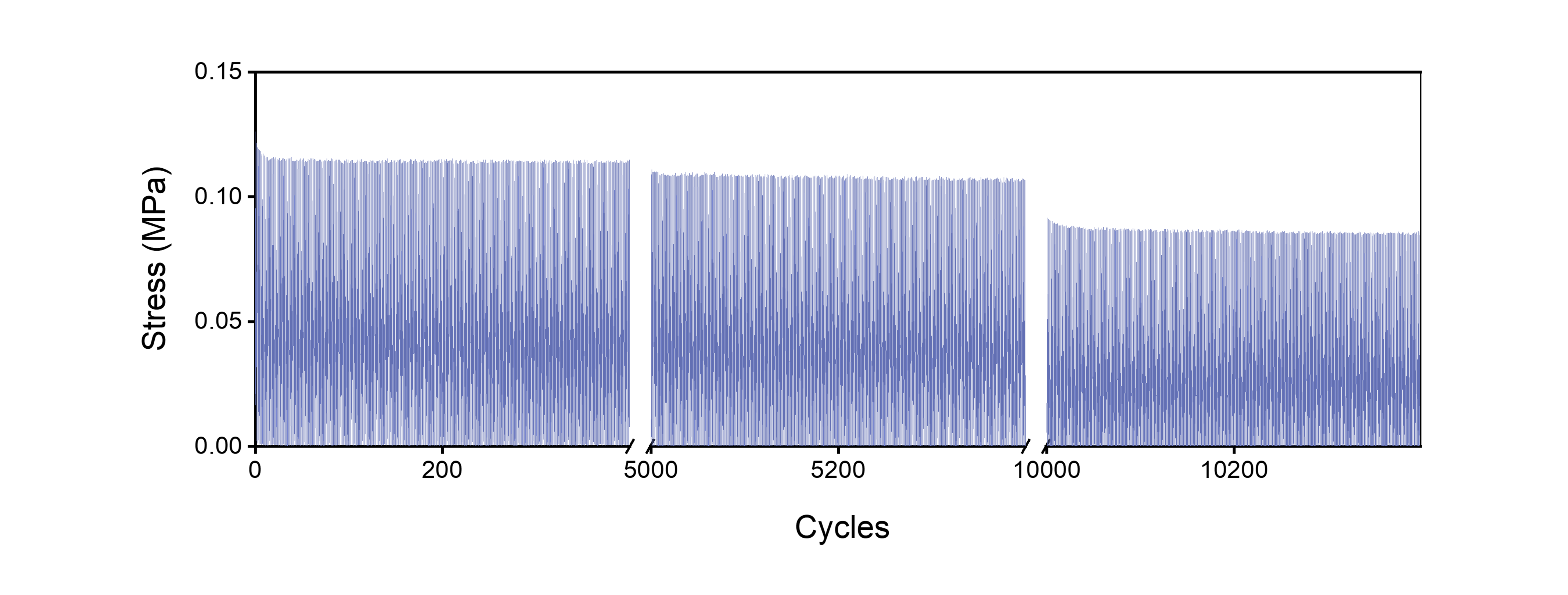


**Fig. S8** Fatigue test of the PAM-ALG-PANI hydrogel under repeated loading-unloading cycles at 30% strain


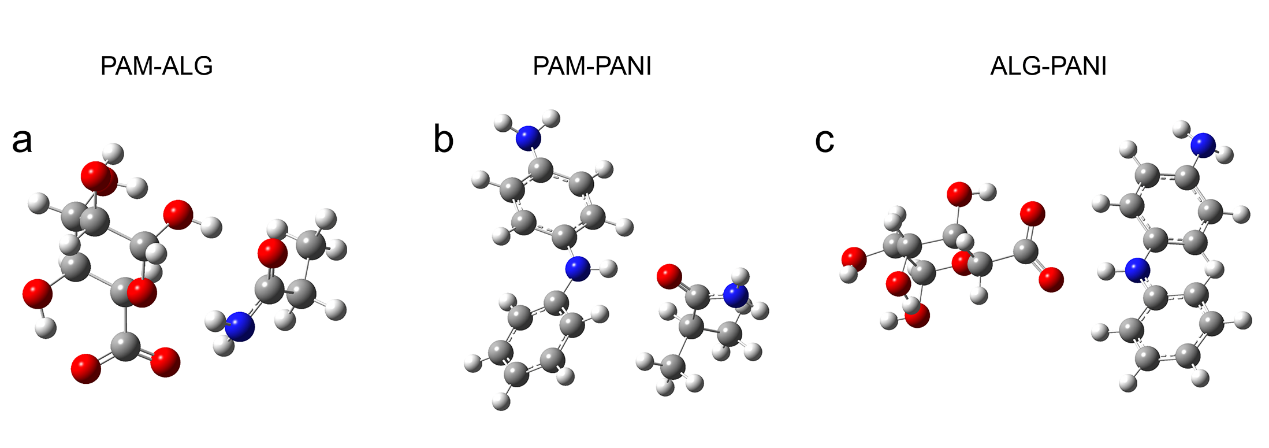


**Fig. S9** DFT simulations of hydrogen bonding in PAM−ALG−PANI hydrogels. **a** Hydrogen bonds between PAM and ALG showed bond lengths of 1.84 Å (O–H···O), 2.05 Å (O···H–N), and 1.95 Å (O(COO⁻)···H–N) with a binding energy of ΔG = −10.1 kcal/mol. **b** Hydrogen bonds between PAM and PANI were characterized by a bond length of 2.02 Å (O···H–N) and ΔG = −65 kcal/mol. **c** For ALG and PANI, hydrogen bonds exhibited a bond length of 1.82 Å (O···H–N) and ΔG = −75.6 kcal/mol


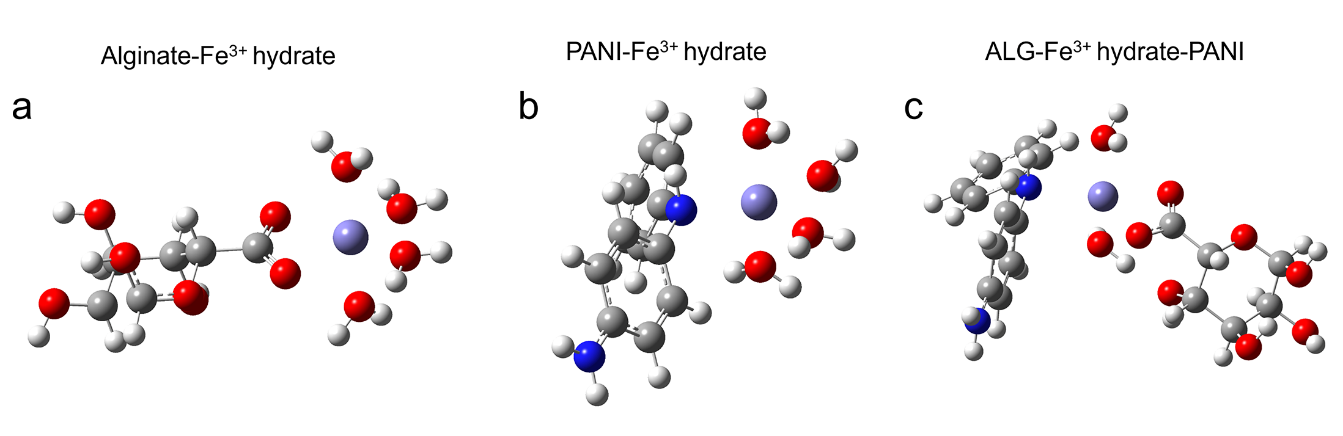


**Fig. S10** DFT simulations of coordination bonding in PAM−ALG−PANI hydrogels. **a** The simulations identified coordination bonds between Alginate and Fe³⁺ hydrate with Fe–O(COO⁻) bond lengths of 2.18 Å and 2.20 Å, accompanied by a binding energy of ΔG = −338.4 kcal/mol. **b** Coordination bonds in PANI−Fe³⁺ hydrate exhibited Fe–N bond lengths of 2.1 Å and ΔG = −164.4 kcal/mol. **c** In the Alginate−PANI−Fe³⁺ hydrate system, coordination bonds displayed Fe–N (2.3 Å) and Fe–O(COO⁻) (2.1 Å, 2.1 Å) bond lengths with ΔG = −424.7 kcal/mol


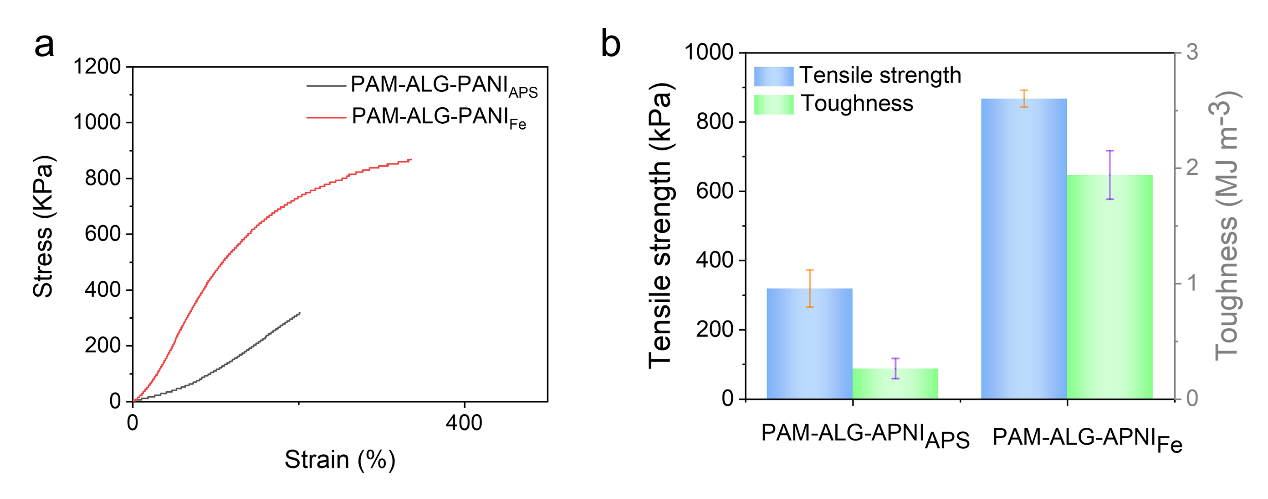


**Fig. S11** Tensile properties of PAM−ALG−PANI hydrogels with different initiators. **a** The tensile stress−strain curves of the APS−initiated PAM−ALG−PANI_APS_ hydrogel and the Fe^3+^ initiated PAM−ALG−PANI_Fe_ hydrogel, and **b** the corresponding tensile strength and toughness


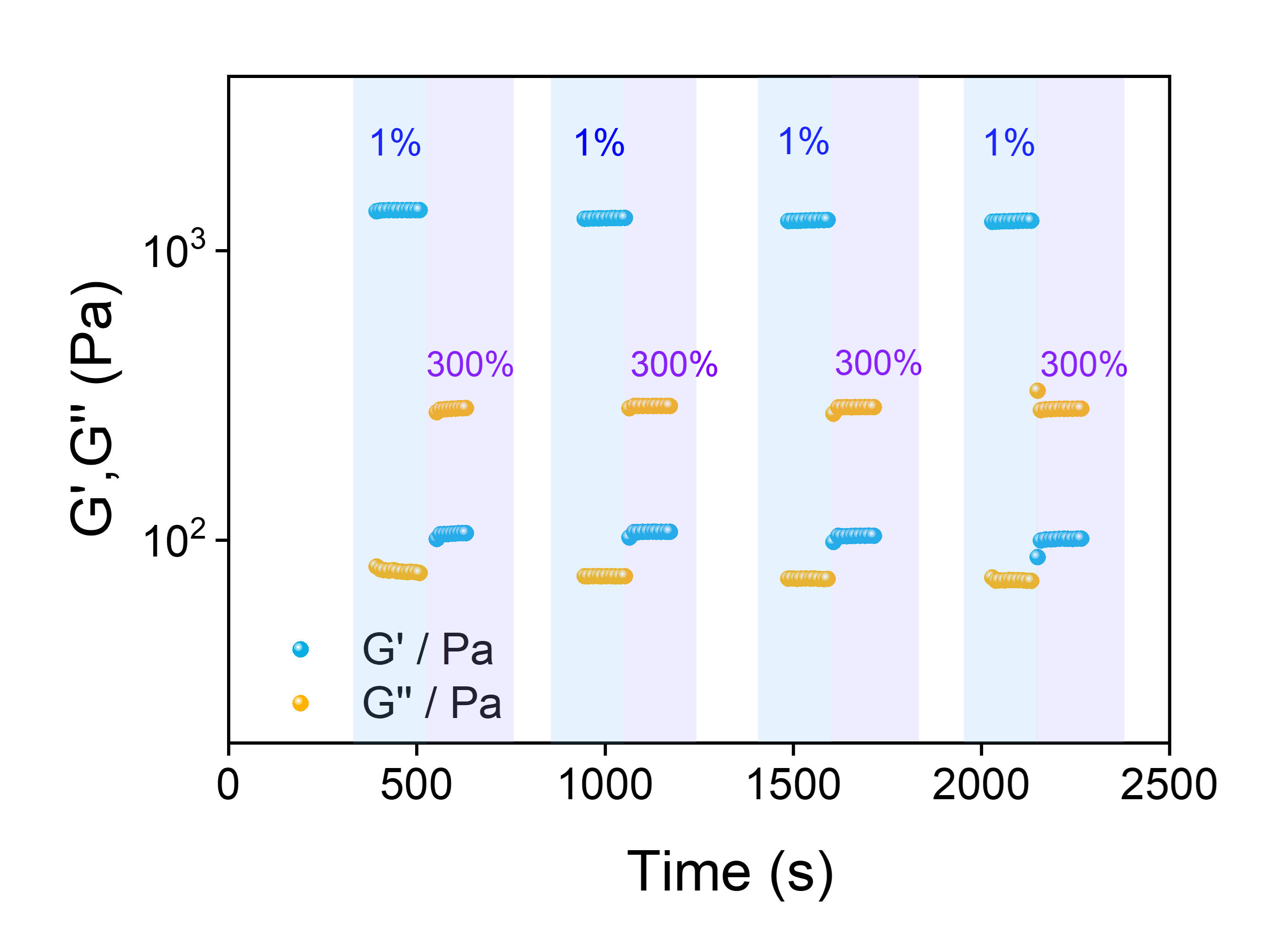


**Fig. S12**. Cyclic shear strain measurement of PAM-ALG-PANI hydrogel switching between small shear strain (1%) and large shear strain (300%). The PAM-ALG-PANI hydrogel was subjected to dynamic oscillatory shear testing by applying alternating cycles of 1% shear strain for 100 s and 300% shear strain for 100 s. Under 1% strain, the storage modulus (G′) consistently exceeded the loss modulus (G″), indicating solid-like elastic behavior. When the strain was increased to 100%, G″ gradually surpassed G′, resulting in a reversal of the moduli relationship. Subsequently, upon reduction of the strain back to 1% and after four recovery periods, G′ recovered to values higher than G″, with the modulus gradually recovering to approximately 91% of its original value, which confirmed that the hydrogel regained its elastic solid behavior. This recoverable transition in mechanical behavior suggests that the dynamic physical cross-linking network within the hydrogel can be restored after the removal of strain.


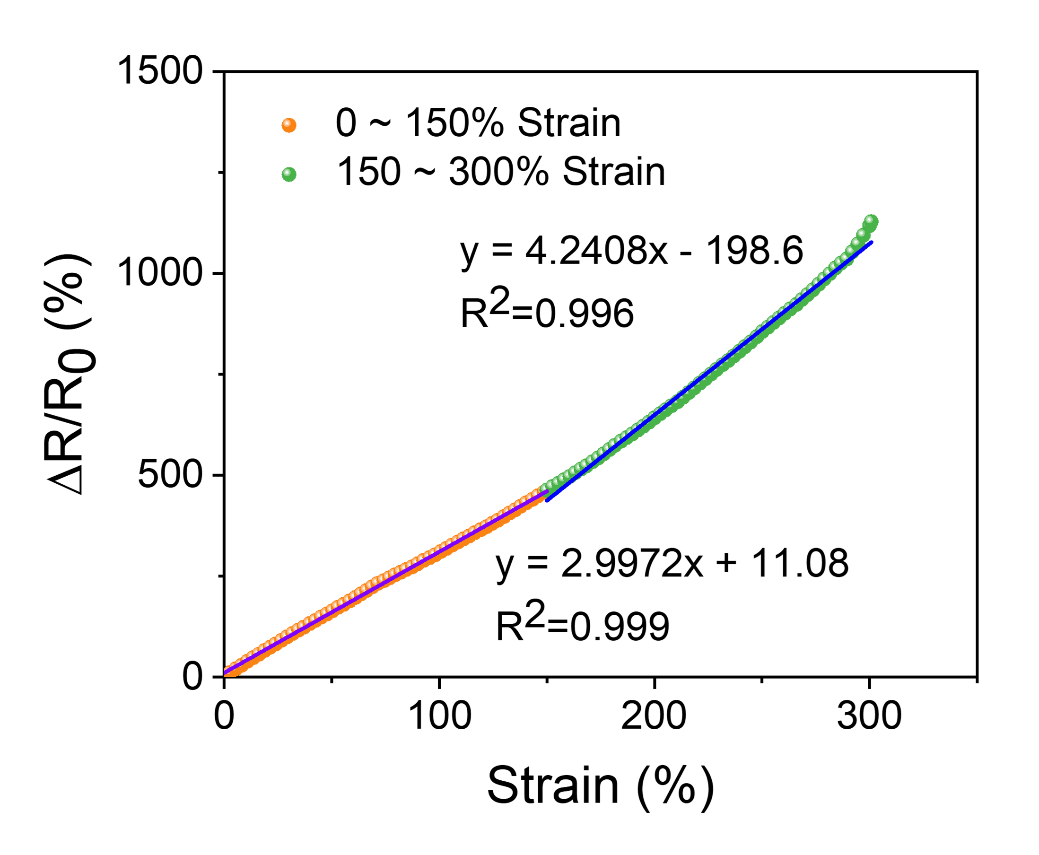


**Fig. S13** The fitting curve and corresponding equation for the relationship between relative resistance change and strain in PAM−ALG−PANI hydrogel. The slope of the relative resistance change (ΔR/R_0_)−strain (ε) curve is defined as the gauge factor (GF), mathematically expressed as: GF = (ΔR/R_0_)/ε × 100%


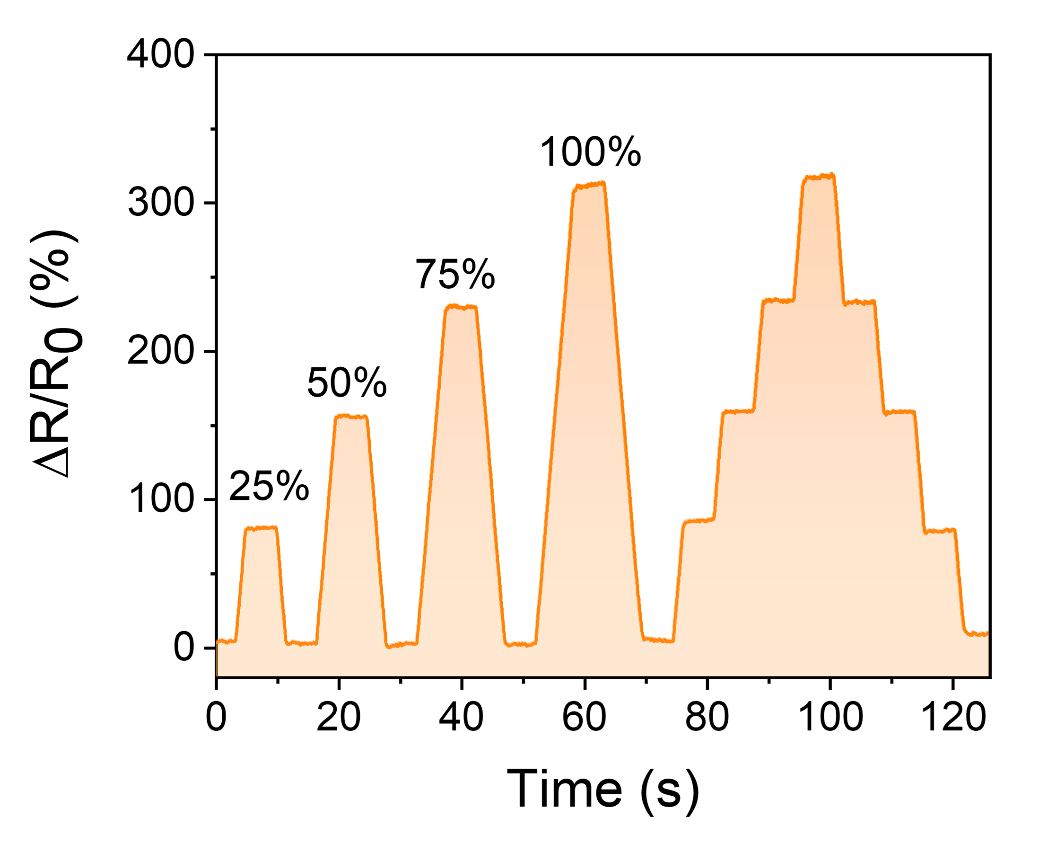


**Fig. S14** Relative resistance change of hydrogel sensors during stepwise loading−unloading from 0% to 100% at 25% per step


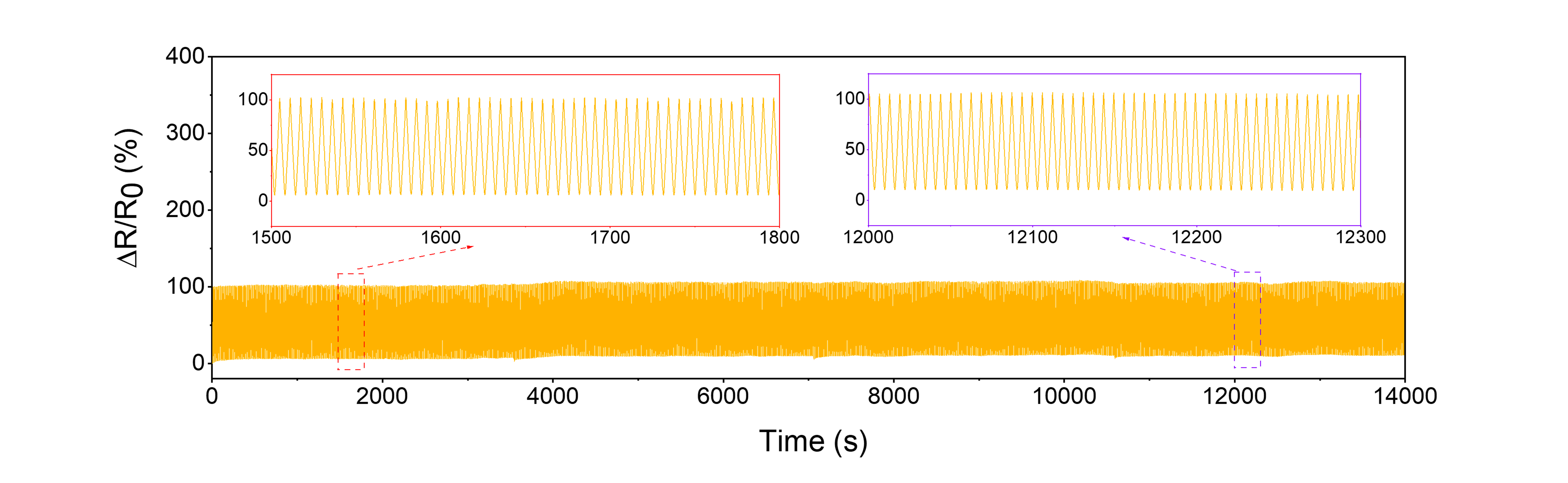


**Fig. S15** Relative resistance change of the PAM-ALG-PANI hydrogel sensor after 2200 loading-unloading cycles, following one week of storage in high-humidity conditions (Room temperature, RH=90%). To ensure measurement accuracy, all electrical tests were performed on the hydrogel in an encapsulated setup to isolate it from ambient conditions


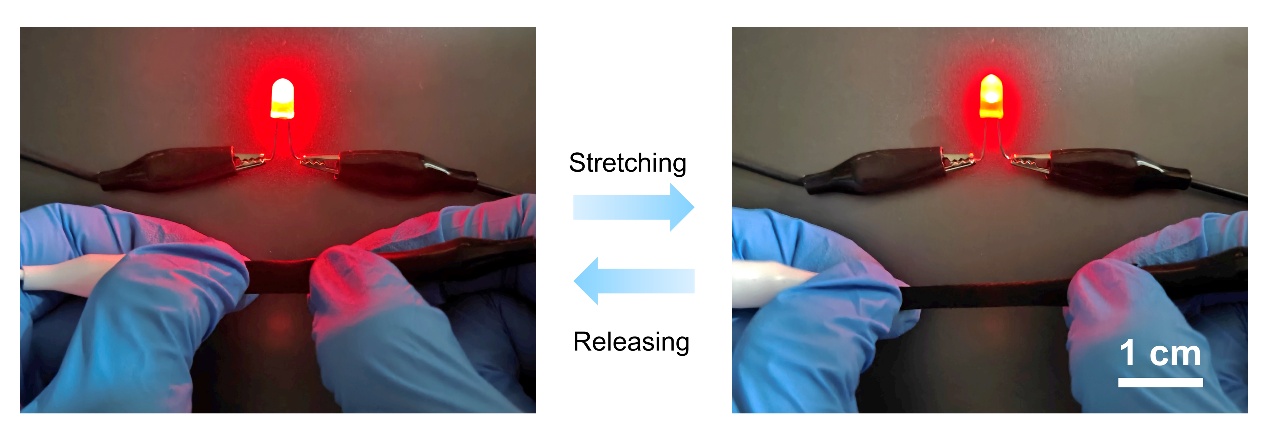


**Fig. S16** Photographs of the bright and dark changes in LEDs during cyclic stretching of the hydrogel


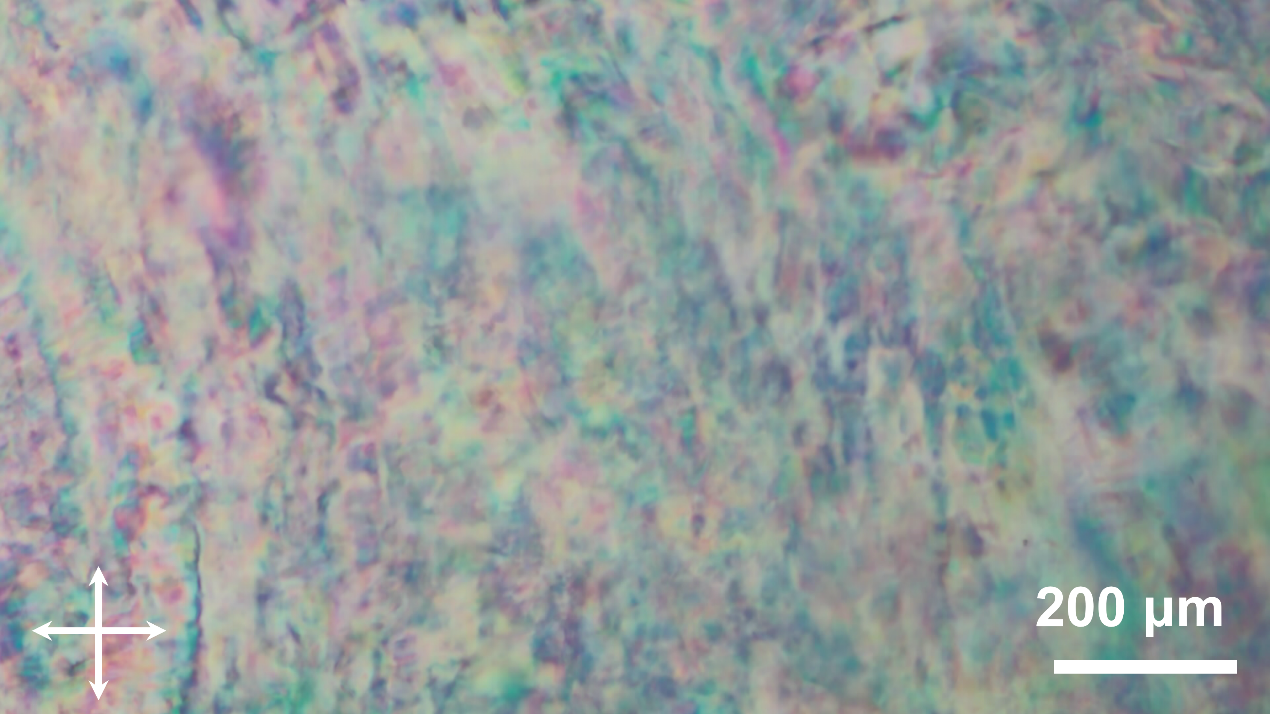


**Fig. S17** Polarized optical microscope picture of HPC


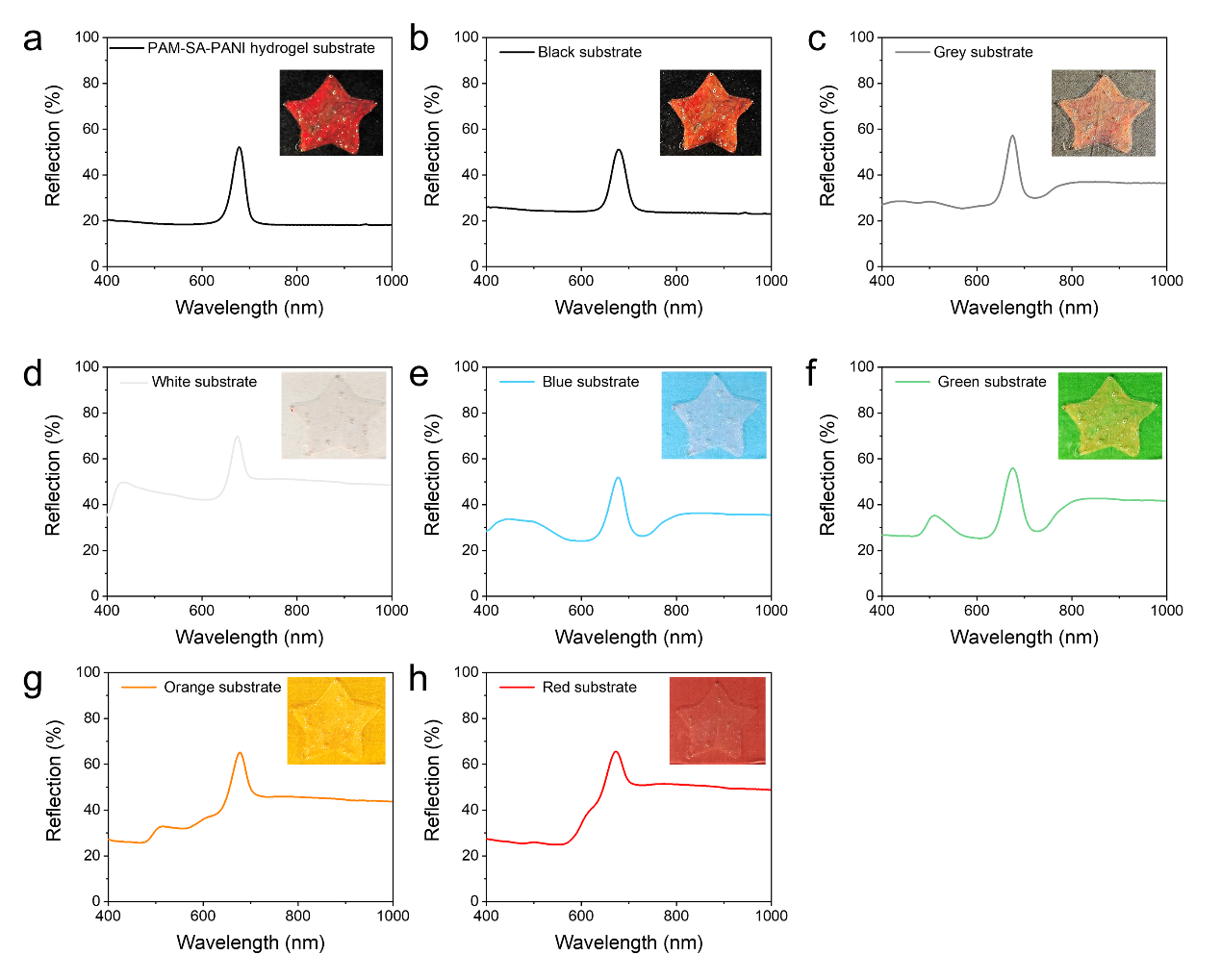


**Fig. S18** Reflectance spectra of composite devices with different colored substrates, including **a** PAM−ALG−PANI black hydrogel, **b** black, **c** grey, **d** white, **e** blue, **f** green, **g** orange, **h** red


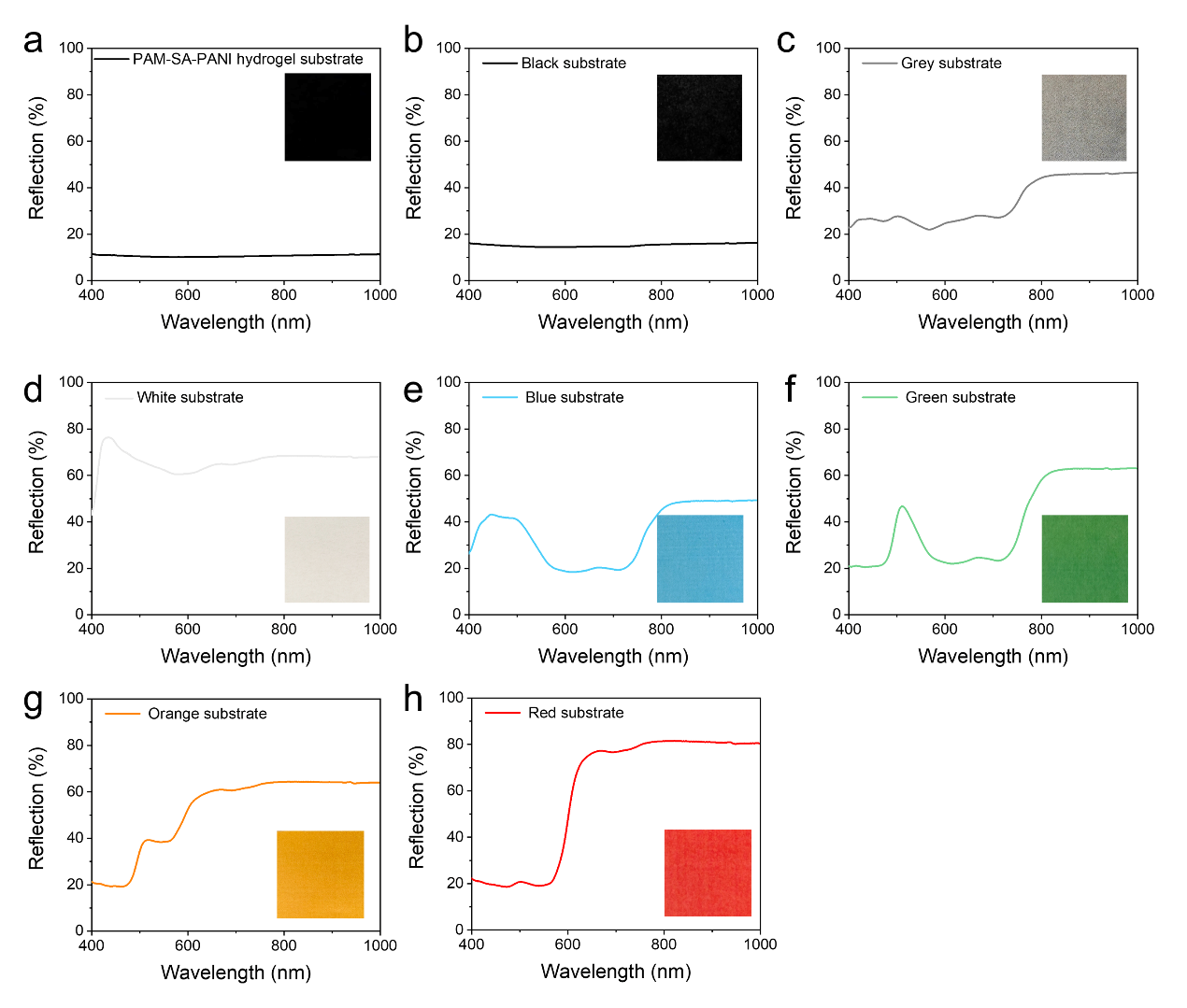


**Fig. S19** Intrinsic reflectance spectra of different colored substrates, including **a** PAM−ALG−PANI black hydrogel, **b** black, **c** grey, **d** white, **e** blue, **f** green, **g** orange, **h** red


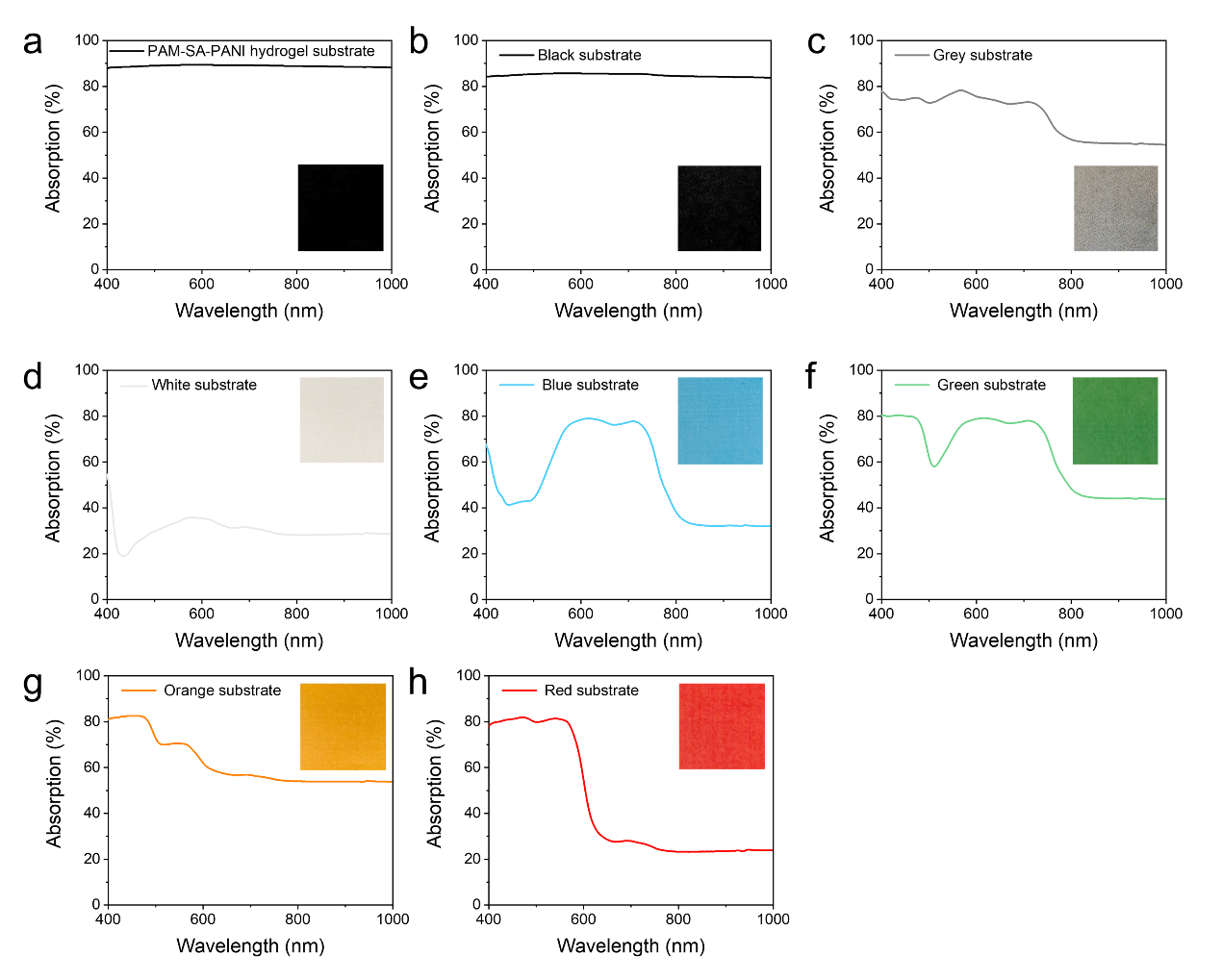


**Fig. S20** Intrinsic absorption spectra of different colored substrates, including **a** PAM−ALG−PANI black hydrogel, **b** black background, **c** grey, **d** white, **e** blue, **f** green, **g** orange, **h** red


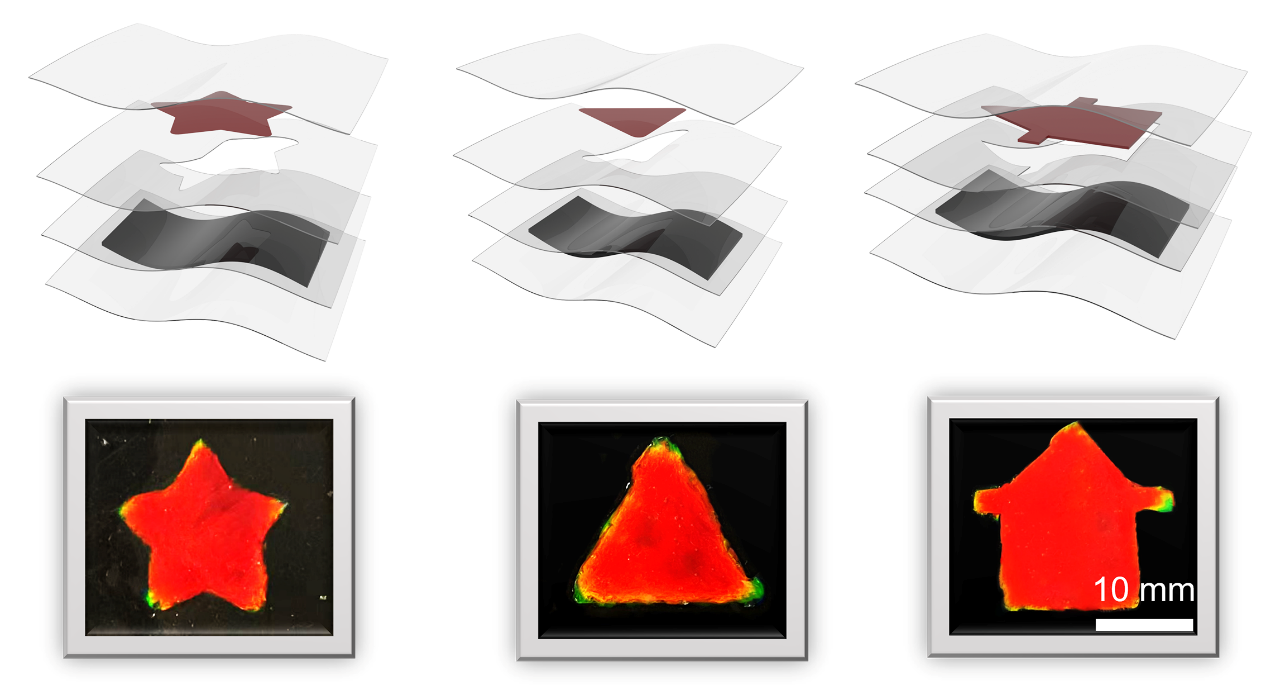


**Fig. S21** The optical images of devices with structurally colored layers in diverse geometries were fabricated through shape modulation of the template layer


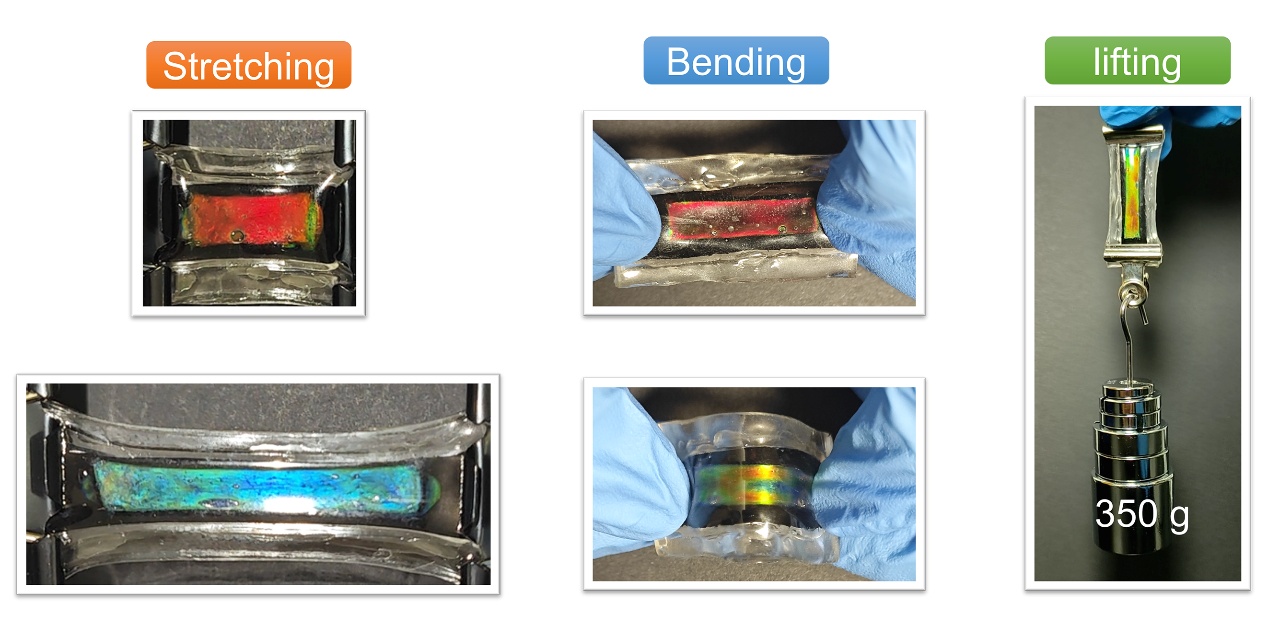


**Fig. S22** Optical images of DM−BCESC sensor demonstrating stretching, bending, and lifting of a 350 g weight with concurrent color changes


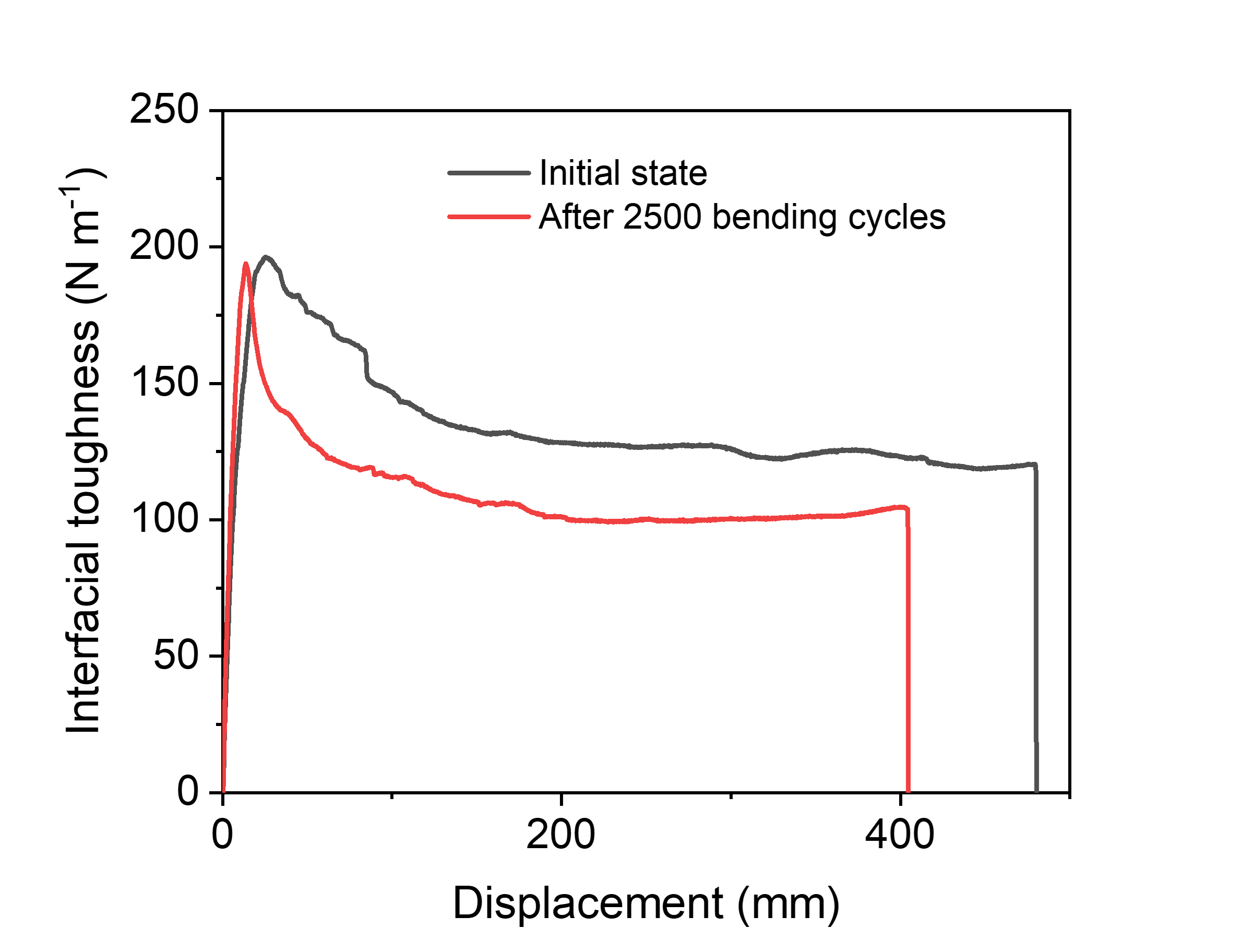


**Fig. S23** The interfacial toughness (calculated as twice the peel force/width) of the VHB layer used for device encapsulation was evaluated via 180° peel tests. The initial device dimensions were 50 mm (length) × 25 mm (width). In the pristine state, the interlayer peel strength of the VHB measured 125.66 N/m. After 2500 bending cycles, the peel strength remained high at 100.35 N/m, corresponding to 80.6% of the initial value. This high retention rate indicates that the VHB encapsulation interface maintains strong adhesion even after prolonged bending cycles, ensuring continued reliable protection for the device


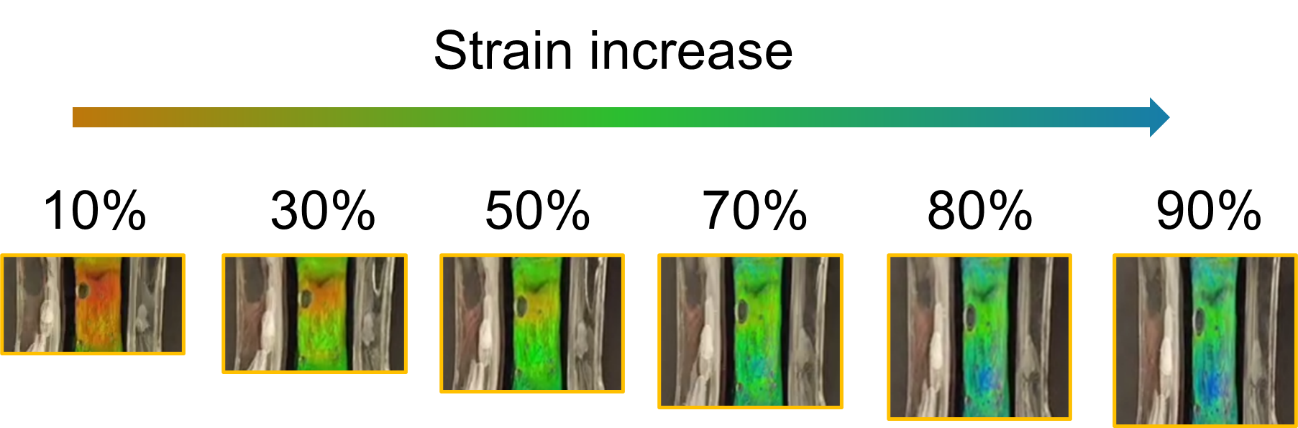


**Fig. S24** Optical images of the sensor’s color change at strain from 10%~90%


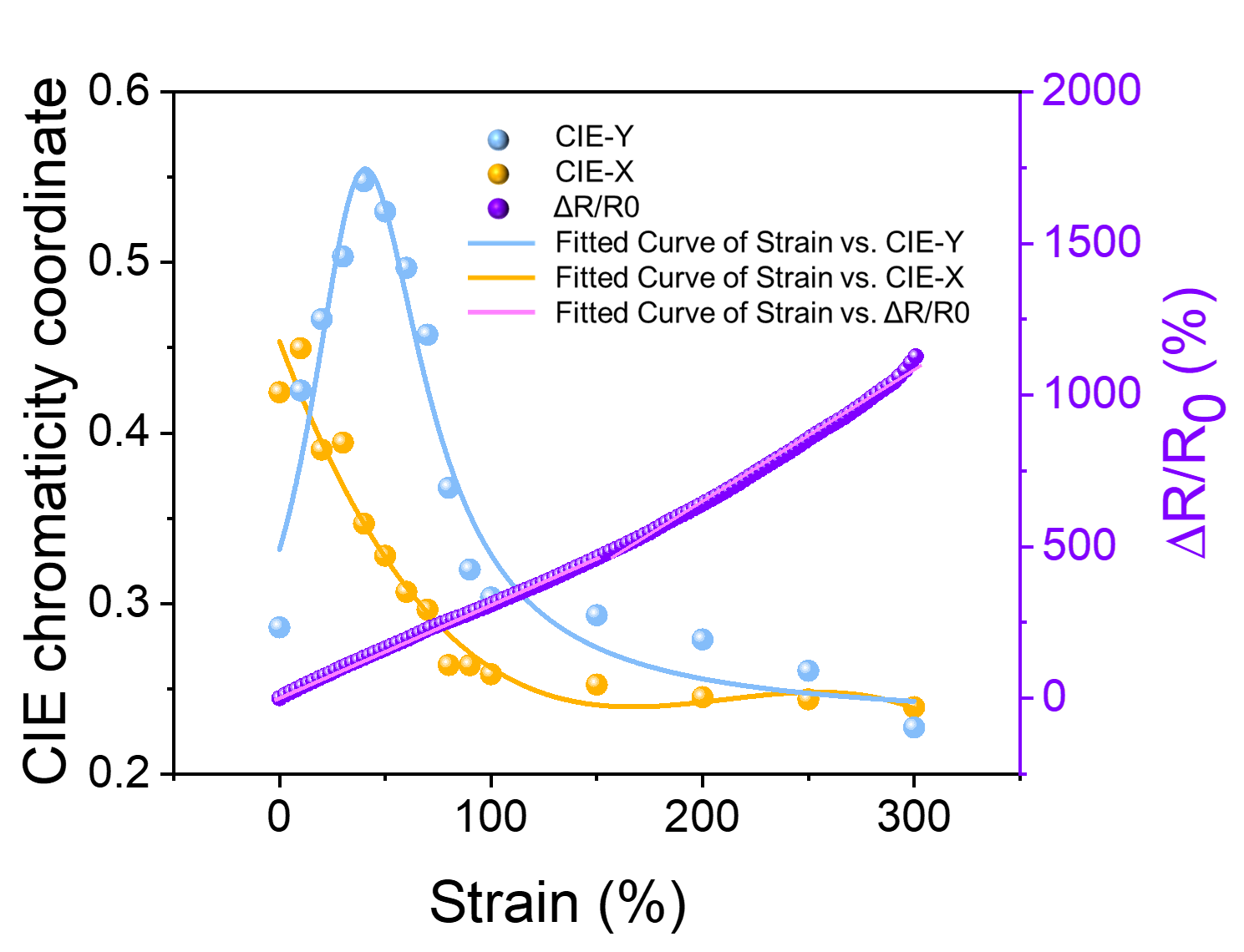


**Fig. S25** Fitted curves of strain, CIE parameters, and relative resistance change rate. The functional relationship between strain (*x*) and CIE-Y (*y*_(CIE-Y)_) is: *y*_(CIE-Y)_ = -1.39566×10^-8^ *x*^3^ + 2.171714×10^-5^*x*^2^ - 0.002864158*x* + 0.46635. The functional relationship for the fitted curve between strain (*x*) and CIE-X (*y*_(CIE-X)_) is given by: *y*_(CIE-X)_ = (4.91872 -0.10738*x*+ 0.00154*x*^2^)/ (13.33492 -0.41395*x* + 0.00546*x*^2^). For the fitted curve between strain (*x*) and relative resistance change rate (*y*_(R)_), the functional relationship is: *y*_(R1)_ = 2.9972*x* + 11.08 (for *x* in 0-150%), and *y*_(R2)_ = 4.2408*x* - 198.6 (for *x* in 150%-300%).


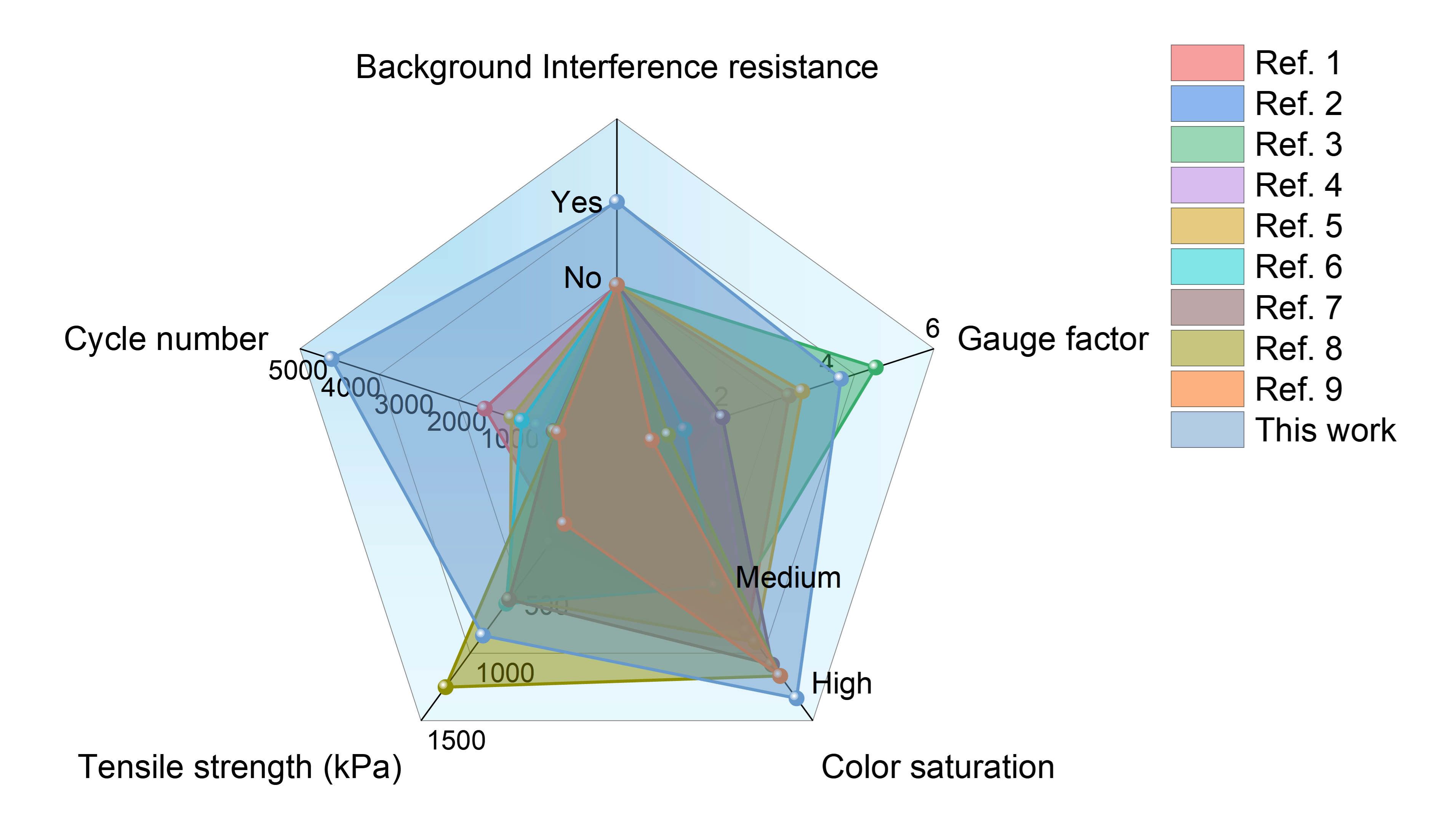


**Fig. S26** Performance comparison with previously reported dual-mode sensors [S1-S9], including background interference resistance, sensitivity (gauge factor), color saturation, tensile strength, and cycle number. Compared with other dual-mode sensing devices reported previously, our designed DM-BCESC sensor exhibits commendable comprehensive performance. Notably, most reported dual-mode sensors have strong background dependence and require a dark background for color observation. In contrast, the dual-mode sensor we developed based on the black CPH-enhanced structural color strategy is background-independent while maintaining high color saturation.


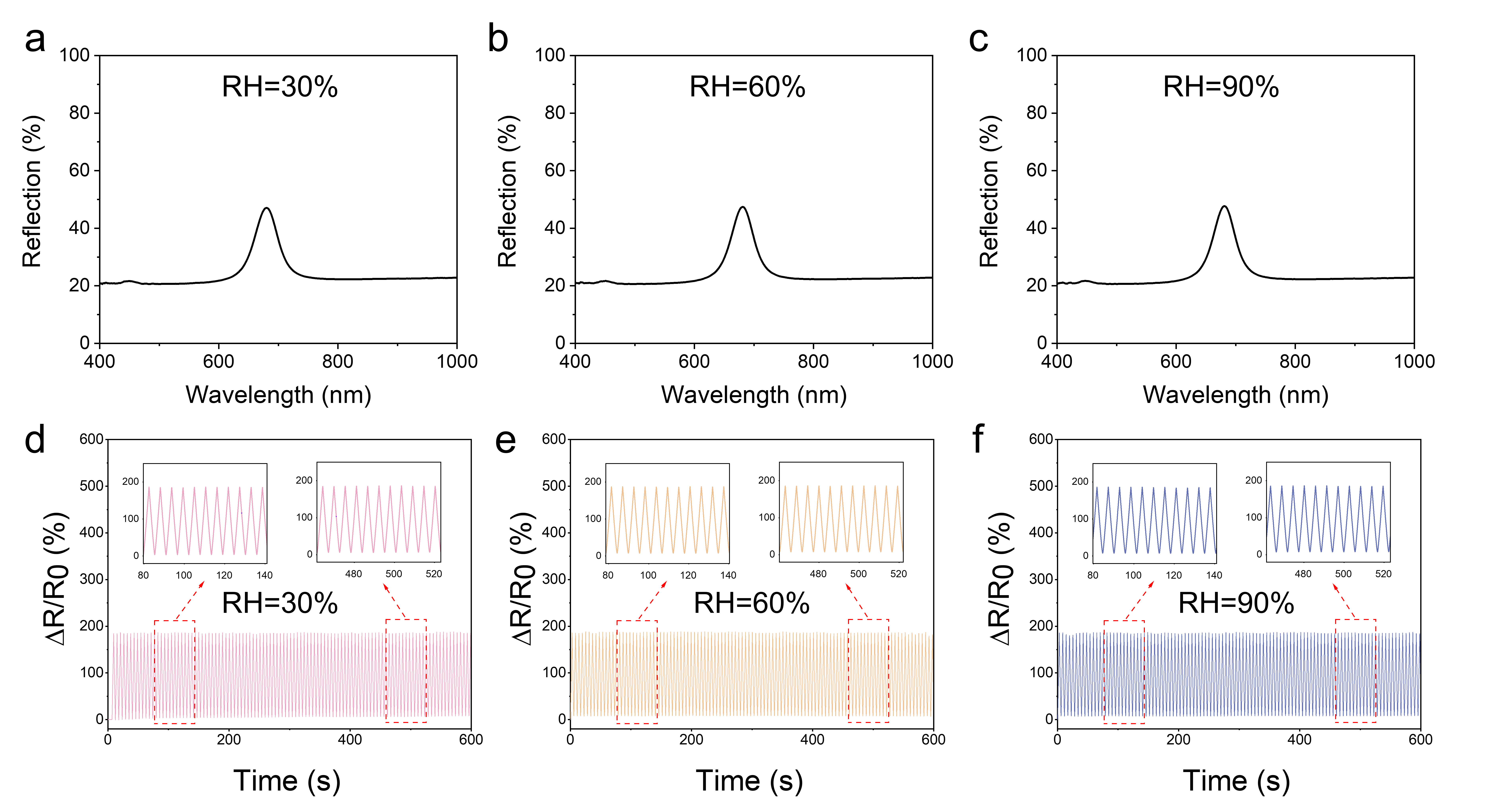


**Fig. S27** Humidity stability test of the DM-BCESC sensor. (**a-c**) Reflection spectra of the sensor’s HPC layer under different humidity levels. (**d-f**) Relative resistance signal changes during loading-unloading cycles (50% strain) under different humidity levels. The sensor’s HPC layer maintains a reflection peak around 680 nm in the visible region across all humidity conditions. Similarly, the PAM-ALG-PANI hydrogel layer delivers a stable signal output without fluctuations under varying humidity. Thus, the sensor demonstrates outstanding humidity stability. It reliably maintains consistent optical and electrical signal outputs in both dry and humid environments, unaffected by air humidity.


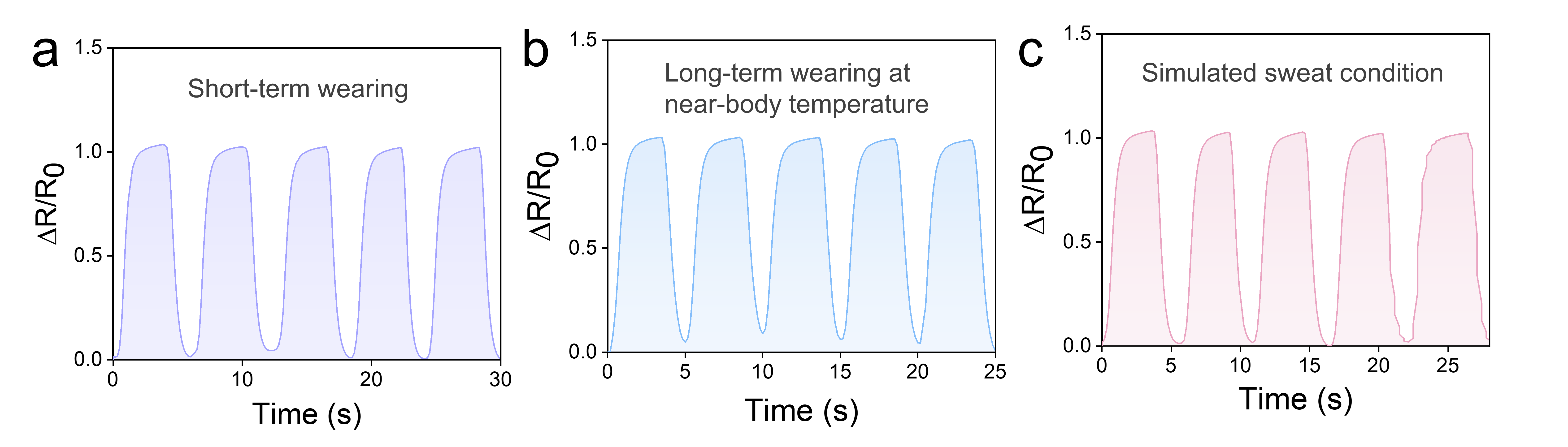


**Fig. S28** Stability testing of sensor resistance signals under different actual wearing conditions. **a** Relative resistance changes in the sensor under short-term wearing conditions. **b** Relative resistance changes detected by the sensor after long-term wearing at near-body temperature. **c** Relative resistance changes captured by the sensor under simulated sweat conditions


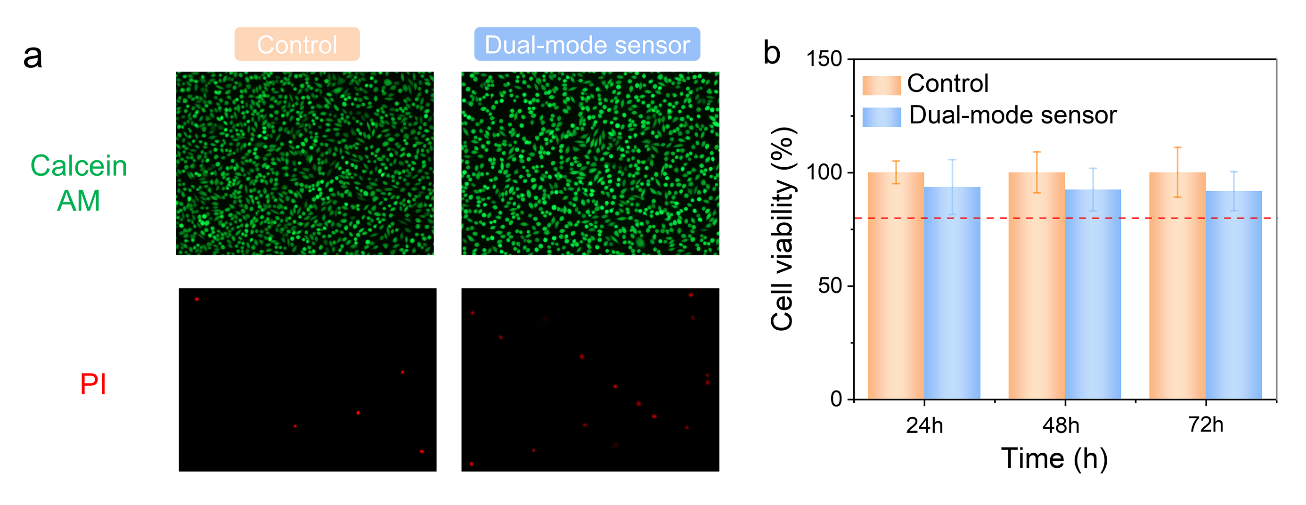


**Fig. S29** Biocompatibility assessment of the dual-mode sensor. **a Calcein-acetoxymethyl / Propidium Iodide (Calcein-AM/PI)** staining of **Human Umbilical Vein Endothelial Cells (HUVECs)** cultured for 24 hours in **Dulbecco’s Modified Eagle’s Medium (DMEM)** leachate obtained after soaking the device for 1 day. Green indicates live cells; red indicates dead cells. The control group used pure DMEM medium. **b** **Cell Counting Kit-8 (CCK-8)** assay data of HUVECs continuously cultured in the device leachate at different time points. Live/dead staining revealed comparable numbers of viable cells in both the dual-mode sensor extract group and the control group after 24 hours, demonstrating the sensor’s biocompatibility. Cytotoxicity testing showed high cell viability rates in the extract group: 93.5 ± 12.1% at 24 hours, 92.4 ± 9.4% at 48 hours, and 91.7 ± 8.6% at 72 hours. These results confirm that the dual-mode sensor exhibits excellent biocompatibility with no significant cytotoxicity, supporting its use for on-body applications.


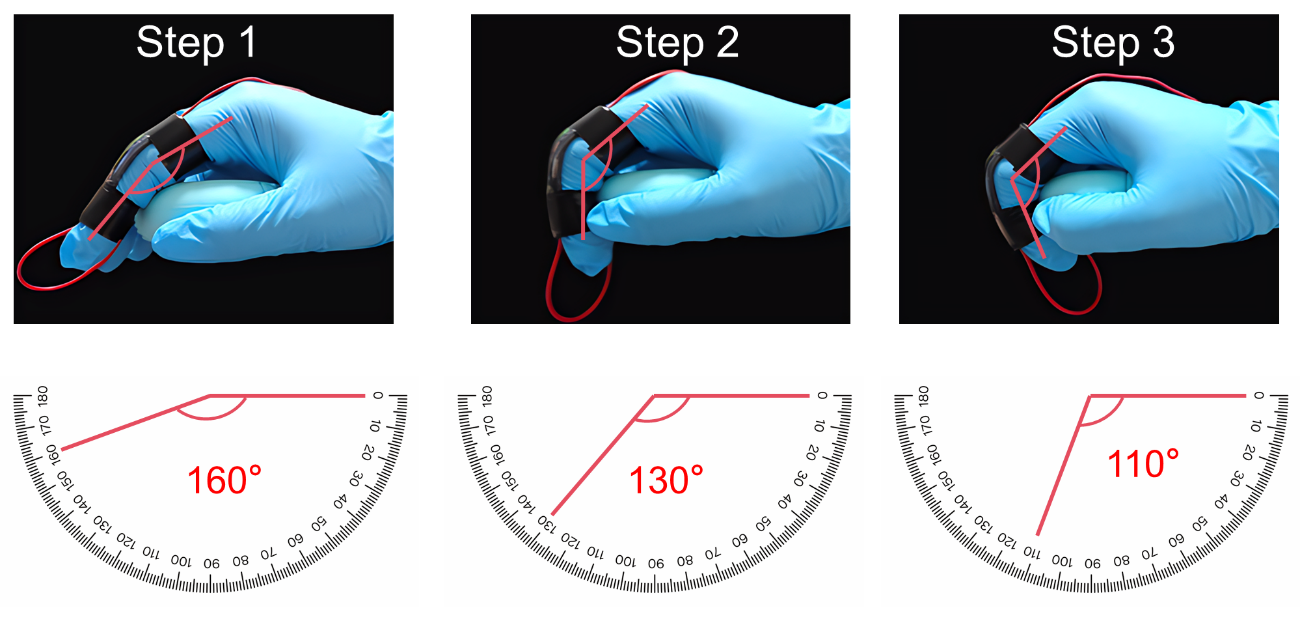


**Fig. S30** Schematic diagram and measurement of flexion angles of finger joints during grip ring training. From step 1 to step 3, with increasing finger bending magnitude, the finger joint angles progressively decrease


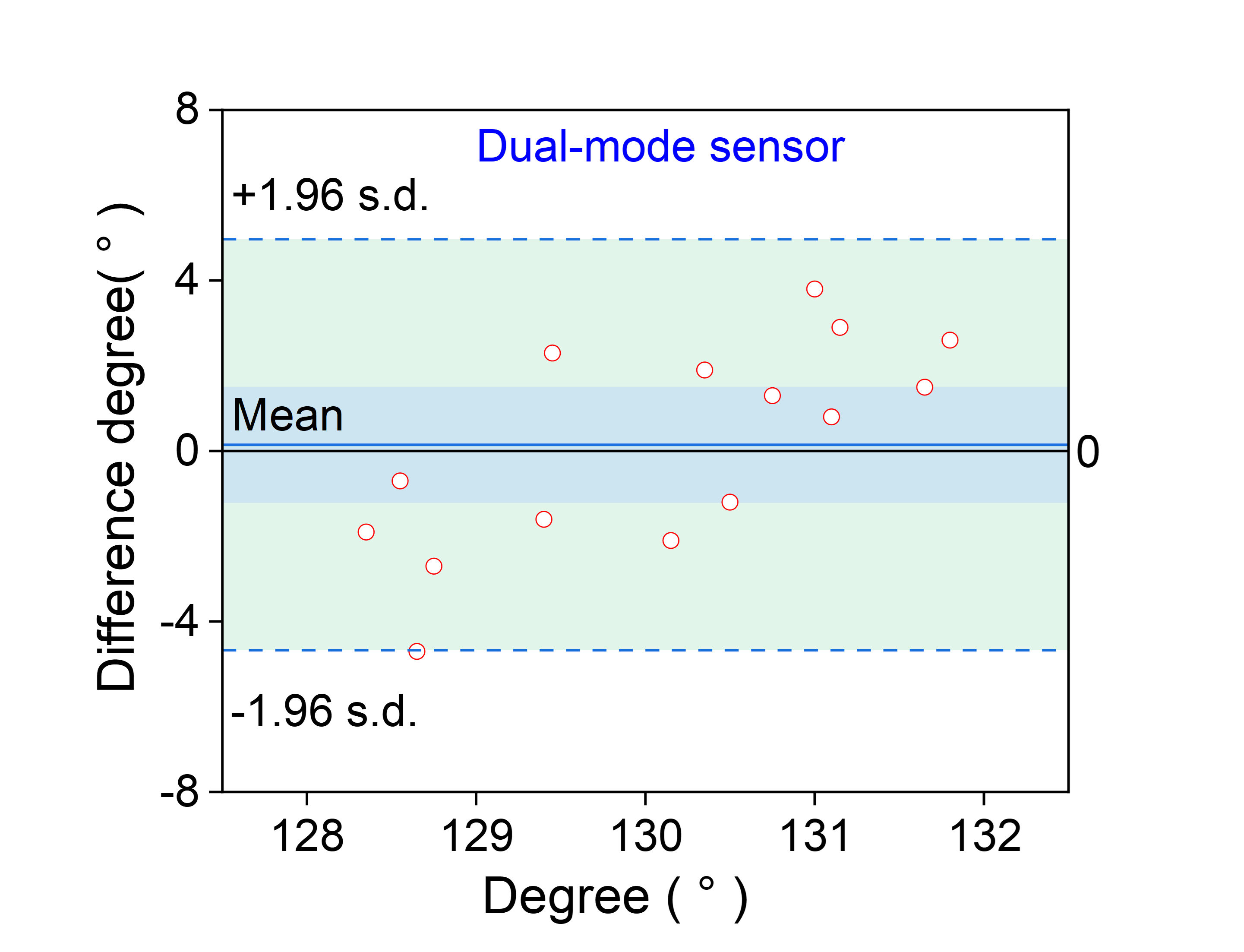


**Fig. S31** Bland-Altman plot comparing joint flexion angle measurements from the dual-mode sensor and a commercial goniometer. The x-axis shows the mean angle calculated from both methods. The y-axis shows the difference between methods (sensor value minus goniometer value). The solid center line represents the mean difference. The upper and lower dashed lines indicate the 95% limits of agreement (LoA), assessing measurement consistency. Bland-Altman analysis revealed a mean difference of 0.15° between the dual-mode sensor and goniometer measurements, which is close to zero. This indicates no significant systematic bias compared to the clinically standard goniometer, validating the sensor’s accuracy and reliability for joint flexion feedback. The 95% LoA ranged from -4.67° to 4.97°, and nearly all 15 data points fell within these limits, further demonstrating strong agreement between the two methods. Therefore, the dual-mode sensor provides reliable measurements. Its mechanochromic response effectively indicates joint flexion degree, offering accurate real-time feedback for movement correction during rehabilitation training.


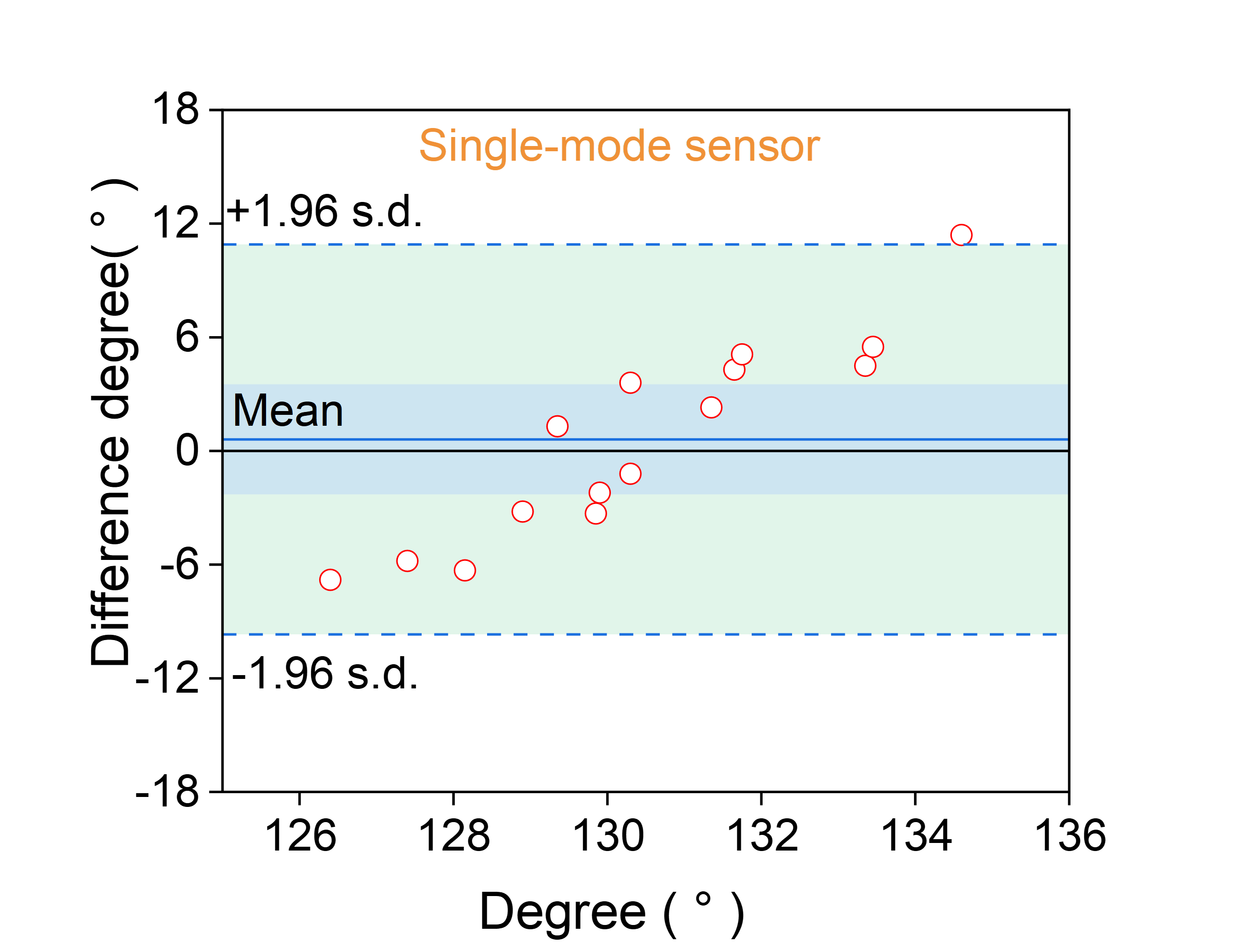


**Fig. S32** Bland-Altman plot for agreement between joint flexion angle measurements from the single-mode resistive sensor and a commercial goniometer. The x-axis represents the mean value from both methods. The y-axis represents the difference between the single-mode sensor and the goniometer measurements. Bland-Altman analysis revealed significantly inferior measurement stability for the single-mode sensor compared to the dual-mode sensor. Its mean bias was 0.61°, which is 4.1 times that of the dual-mode sensor (0.15°). The 95% LoA ranged from -9.68° to 10.90°, yielding an interval width of 20.58°. This width is 2.1 times wider than the dual-mode sensor’s LoA (9.64°). Furthermore, the single-mode sensor exhibited maximum positive and negative differences as high as +11.4° and -6.8°, respectively, exceeding the dual-mode sensor’s range of ± 4.7°. These results indicate that the single-mode sensor exhibits not only a larger overall measurement bias but also significantly wider variability in individual data points, demonstrating markedly inferior stability. The single-mode resistive sensor relies solely on electrical signals for angle output, requiring external devices for signal processing and display. Consequently, users cannot directly perceive joint flexion angles during rehabilitation training. This introduces delay and barriers in accessing feedback, hindering the rapid assessment of movement compliance with training standards. In contrast, the dual-mode sensor overcomes the real-time feedback limitation inherent in single-mode designs. It enables users to visually assess movement accuracy via direct color cues, significantly enhancing interaction efficiency and accuracy during rehabilitation training.


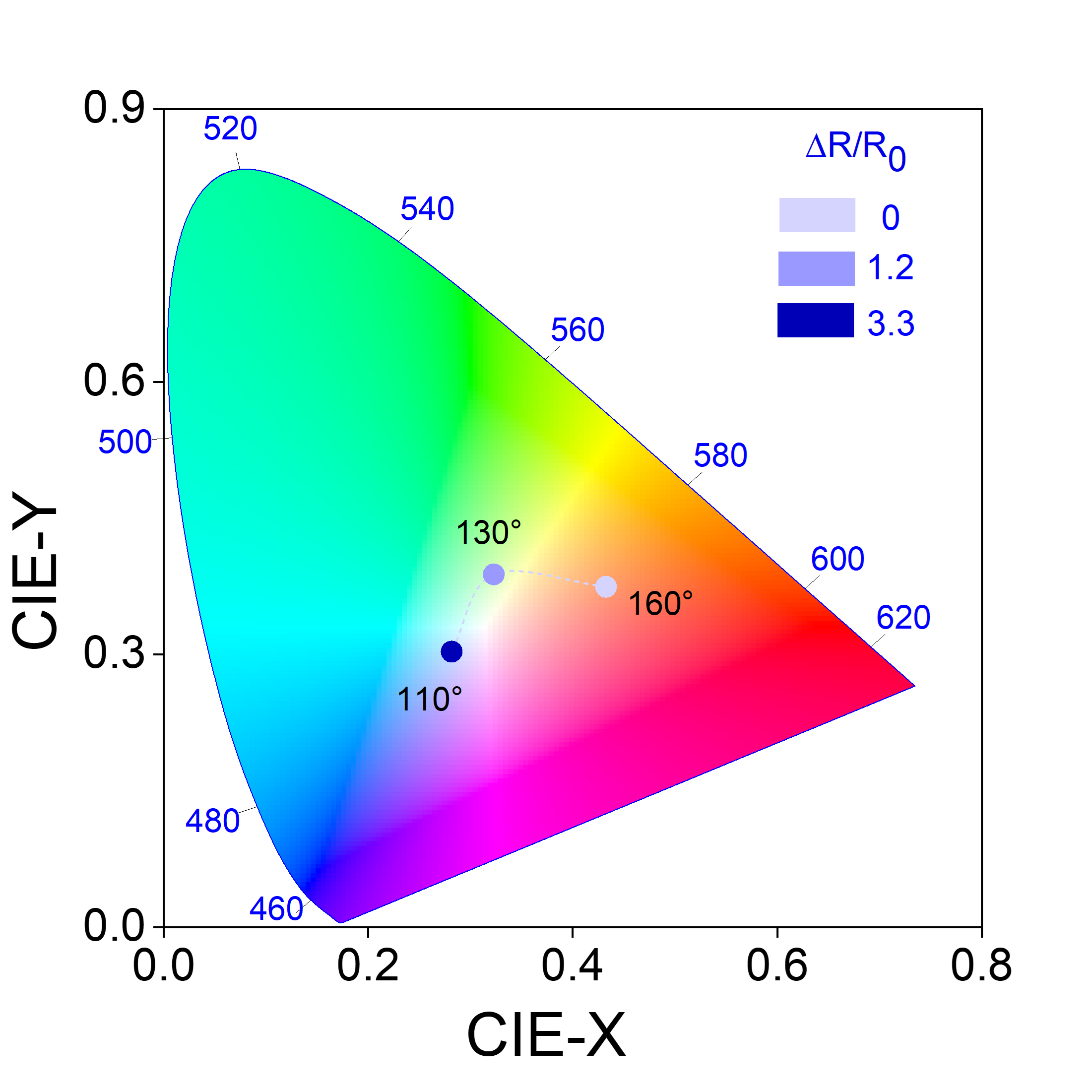


**Fig. S33** During grip ring training, the sensor captures both the CIE color coordinates and resistance signals corresponding to the finger’s bending angle. The sensor’s color feedback falls within the red, green, and blue regions of the spectrum, which are colors clearly distinguishable to the human eye. Furthermore, as the finger’s bending amplitude increases (the joint angle decreases), the sensor simultaneously detects correspondingly larger and distinguishable resistance signals.

**Supplementary References**

1. Y. Shang, C. Huang, Z. Li, X. Du, Bioinspired ultra-stretchable and highly sensitive structural color electronic skins. Adv. Funct. Mater. **35**(2), 2412703 (2025). <https://doi.org/10.1002/adfm.202412703>
2. X. Hu, J. Wang, S. Song, W. Gan, W. Li et al., Ionic conductive konjac glucomannan/liquid crystal cellulose composite hydrogels with dual sensing of photo- and electro-signals capacities as wearable strain sensors. Int. J. Biol. Macromol. **258**, 129038 (2024). <https://doi.org/10.1016/j.ijbiomac.2023.129038>
3. L. Bai, Y. Jin, X. Shang, H. Jin, Y. Zhou et al., Highly synergistic, electromechanical and mechanochromic dual-sensing ionic skin with multiple monitoring, antibacterial, self-healing, and anti-freezing functions. J. Mater. Chem. A **9**(42), 23916–23928 (2021). <https://doi.org/10.1039/D1TA06798B>
4. R. Zhao, Y. He, Y. He, Z. Li, M. Chen et al., Dual-mode fiber strain sensor based on mechanochromic photonic crystal and transparent conductive elastomer for human motion detection. ACS Appl. Mater. Interfaces **15**(12), 16063–16071 (2023). <https://doi.org/10.1021/acsami.3c00419>
5. X. Li, J. Liu, Q. Guo, X. Zhang, M. Tian, Polymerizable deep eutectic solvent-based skin-like elastomers with dynamic schemochrome and self-healing ability. Small **18**(19), 2201012 (2022). <https://doi.org/10.1002/smll.202201012>
6. M. Zhao, P. Ren, Q. Lyu, X. Chen, H. Wang et al., Adhesive photonic-ionic skins for visualizing wearable strain distributions. Chem. Eng. J. **470**, 143937 (2023). <https://doi.org/10.1016/j.cej.2023.143937>
7. J. Ma, Y. Yang, X. Zhang, P. Xue, C. Valenzuela et al., Mechanochromic and ionic conductive cholesteric liquid crystal elastomers for biomechanical monitoring and human–machine interaction. Mater. Horiz. **11**(1), 217–226 (2024). <https://doi.org/10.1039/D3MH01386C>
8. X. Li, Y. Yang, C. Valenzuela, X. Zhang, P. Xue et al., Mechanochromic and conductive chiral nematic nanostructured film for bioinspired ionic skins. ACS Nano **17**(13), 12829–12841 (2023). <https://doi.org/10.1021/acsnano.3c04199>
9. W. Li, J. Li, X. Ding, Q. Tan, W. Sun et al., Multi-bioinspired electronic skins with on-demand adhesion and opto-electronic synergistic display capabilities. Innovation **6**(5), 100877 (2025). <https://doi.org/10.1016/j.xinn.2025.100877>
